# YOD1 protects against MRSA sepsis-induced DIC through Lys33-linked deubiquitination of NLRP3

**DOI:** 10.1038/s41419-024-06731-5

**Published:** 2024-05-24

**Authors:** Chang Liu, Caihong Fan, Jia Liu, Shiqi Zhang, Huixin Tang, Yashan Liu, Shengzheng Zhang, Qiang Wu, Jiandong Zhang, Zhi Qi, Yanna Shen

**Affiliations:** 1 School of Medical Technology, Tianjin Medical University, Tianjin, China; 2 Key Laboratory of Emergency and Trauma of Ministry of Education, Hainan Medical University, Haikou, China; 3 Department of Molecular Pharmacology, School of Medicine, Nankai University, Tianjin, China; 4 The Third Central Hospital of Tianjin, Tianjin, China; 5 Institute of Digestive Disease, Shengli Oilfield Central Hospital, Dongying, China; 6 Tianjin Key Laboratory of General Surgery in Construction, Tianjin Union Medical Center, Tianjin, China; 7 The First Department of Critical Care Medicine, The First Affiliated Hospital of Shihezi University, Shihezi, China

**Keywords:** Ubiquitylation, Infection

## Abstract

Disseminated intravascular coagulation (DIC) is considered to be the most common and lethal complication of sepsis. NLR-family pyrin domain-containing-3 (NLRP3) inflammasome plays an important role in host defense against microbial pathogens, and its deregulation may cause coagulation cascade and should be strictly managed. Here, we identified the deubiquitinase YOD1, which played a vital role in regulating coagulation in a NLRP3 inflammasome-dependent manner in sepsis induced by methicillin-resistant *Staphylococcus aureus* (MRSA). YOD1 interacted with NLRP3 to remove K33-linked ubiquitination of NLRP3 based on its deubiquitinating enzyme activity and specifically inhibited expression of NLRP3 as well as activation of NLRP3 inflammasome. Deficiency of YOD1 expression enhanced NLRP3 inflammasome activation and coagulation both in vitro and in vivo. In addition, pharmacological inhibition of the NLRP3 effectively improved coagulation and alleviated organ injury in *Yod1*^*−/−*^ mice infected with MRSA. Thus, our study reported that YOD1 is a key regulator of coagulation during MRSA infection, and provided YOD1 as a potential therapeutic target for the treatment of NLRP3 inflammasome-related diseases, especially MRSA sepsis-induced DIC.

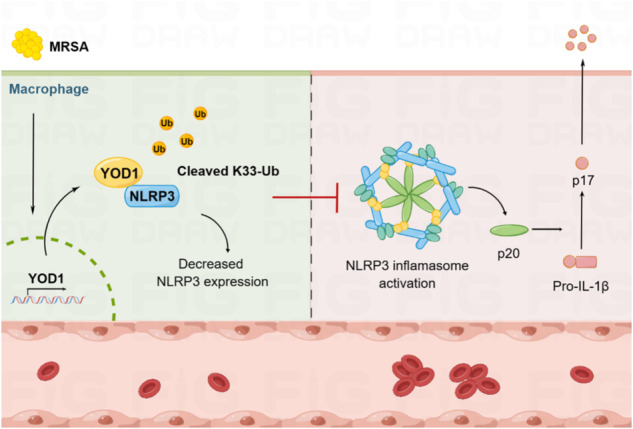

## Introduction

Sepsis is a challenging syndromic response to infection, which has been recognized as the final road to death from many infectious diseases worldwide [1, 2]. According to the World Health Organization, there are 48.9 million cases of sepsis and 11 million sepsis-related deaths worldwide, accounting for nearly 20% of total global deaths [3]. The activation of coagulation and inflammation are essential reactions for host defense during sepsis [4, 5]. Excessive activation of coagulation in sepsis leads to disseminated intravascular coagulation (DIC), a life-threatening complication of sepsis that can result in multiple organ dysfunction syndrome and even death [6]. Notably, the prevalence of DIC in septic patients in the ICU is approximately 50.9%, and the mortality rate in septic patients with DIC is twice as high as that in patients without DIC [7]. Therefore, early diagnosis and treatment are particularly important to improve the survival of patients with sepsis-related DIC.

Macrophages, as the central innate immune cells, are critical for the host against pathogen infection and trigger coagulant responses by recognizing pathogen-associated molecular patterns (PAMPs) through pattern recognition receptors (PRRs), including nucleotide-binding oligomerization domain (NOD)-like receptors (NLRs), retinoic acid-inducible gene I (RIG-I)-like receptors (RLRs), and Toll-like receptors (TLRs) [8–11]. NLR-family pyrin domain-containing-3 (NLRP3), an important member of the NLR family, plays a critical role in mediating the assembly and activation of inflammasome. Once activated, NLRP3 recruits the adapter apoptosis-associated speck like protein containing a CARD (ASC) to bind the effector pro-caspase-1, forming NLRP3 inflammasome. Subsequently, pro-caspase-1 is self-cleaved to active caspase-1, promoting the maturation and secretion of interleukin (IL)-1β and IL-18, as well as gasdermin D (GSDMD)-mediated pyroptosis [8, 9, 12]. The NLRP3 inflammasome promotes the immune system to fight microbial infection, however, aberrant NLRP3 inflammasome activation can contribute to the pathogenesis of several autoinflammatory disorders, cancer, metabolic, and aging-related diseases [13–16]. In addition, the NLRP3 inflammasome is important in endotoxin-induced thrombus formation [17]. Therefore, the activation and stability of NLRP3 inflammasome should be strictly managed.

In resting macrophages, NLRP3 is autoinhibited [18–20]. Upon bacterial infection, NLRP3 undergoes different types of ubiquitination and thus has different outcomes. For instance, several E3 ubiquitination ligases including MARCH7, TRIM31 and Cb1-b mediate K48-linked ubiquitination and degradation of NLRP3, thereby exerting a negative regulation on NLRP3 inflammasome activation [21–23]. Similarly, E3 ubiquitination ligase β-TrCP1 also promotes the proteasomal degradation of NLRP3 via K27-linked ubiquitination [24]. Unlike the preceding ubiquitination, E3 ubiquitination ligase Pellino-2 is responsible for the K63-linked ubiquitination of NLRP3 and promotes the activation of NLRP3 inflammasome [25]. Since ubiquitination is a reversible process, deubiquitinases also play a crucial role in regulating NLRP3 expression and activation. BRCC3 is the first deubiquitinase of NLRP3 essential for NLRP3 inflammasome activation [26, 27]. UAF1/USP1, a deubiquitinase complex, stabilizes NLRP3 by removing its K48-linked ubiquitination [28]. Furthermore, the knockout of STAMBP maintains the K63-linked ubiquitination of NLRP3, which results in faster assembly and greater activity of the inflammasome [29]. However, the deubiquitinases responsible for regulating the activation of NLRP3 inflammasome remain largely unclear.

YOD1 is a highly conserved deubiquitinase of the ovarian tumor proteases (OTU) family, and its biological function, especially in innate immune response, remains largely unclear [30, 31]. It has been reported that YOD1 inhibits neural precursor cell proliferation by targeting neural precursor cell expressed developmentally downregulated protein 4 (NEDD4) for deubiquitination [32]. Suppression of YOD1 prolongs the survival time of acute promyelocytic leukemia cell-bearing mice by promoting the degradation of PML/RARα [33]. Our previous studies have demonstrated that YOD1 negatively regulated RIG-I-mitochondrial antiviral signaling protein (MAVS) signaling during RNA virus infection. YOD1 suppressed MAVS aggregation through cleaving the K63-linked ubiquitination, which led to reduced IFN-β production and cellular antiviral response [34]. However, it remains unknown how YOD1 affects NLRP3 inflammasome-induced DIC. In this study, we identified that YOD1 acted as a critical regulator for coagulation. Mechanically, upon methicillin-resistant *Staphylococcus aureus* (MRSA) infection in macrophages, YOD1 interacted with NLRP3 to specifically remove its K33-linked ubiquitination, subsequently inhibiting NLRP3 inflammasome activation and coagulation. Furthermore, YOD1 protected hosts from the lethal MRSA infection in the mouse model by inhibiting coagulation. Thus, our findings for the first time revealed the immunological role of YOD1 in regulating NLRP3 inflammasome activation and coagulation in MRSA sepsis, and also provided a potential therapeutic target to treat sepsis-induced DIC.

## Materials and methods

### Animal experiments

Male *Yod1* knockout mice on the C57BL/6J background were purchased from Shanghai Model Organisms (Shanghai, China). Wild-type (WT) C57BL/6J mice were purchased from Sipeifu (Beijing, China). To establish an in vivo model of sepsis-induced DIC, mice were randomly assigned to experimental groups. Adult male mice (8 weeks old) were injected with MRSA (1 × 10^8^ colony-forming units/mouse) through a caudal vein. The control group was injected with an equal volume of phosphate buffer solution (PBS). *Yod1*^*−/−*^ mice and their matched WT mice were sacrificed and subjected to coagulation analysis at 12 h after injection of MRSA. All mice were housed in the SPF animal room with free access to water and food.

### Reagents

Anti-NLRP3 (15101), anti-ASC (67824), and anti-Ub (3936) were purchased from Cell Signaling Technology (MA, USA). Anti-IL-1β (ab9722) was purchased from Abcam (Cambridge, UK). Anti-Myc (AE070), anti-HA (AE008), anti-Flag (AE004), anti-Caspase-1 (A0964) and anti-YOD1 (A13270) were purchased from ABclonal (Wuhan, China). Anti-β-actin (T0022) was purchased from Affinity Biosciences (State of Ohio, USA). Protein A/G agarose used for immunoprecipitation was purchased from Thermo Fisher Scientific (Waltham, USA). Mouse D-Dimer ELISA Kit (E-EL-M0400c), Mouse thrombin-antithrombin (TAT) ELISA Kit (E-EL-M1138c), Mouse interleukin 1 Beta (IL-1β) ELISA Kit (E-EL-M0037c), Mouse plasminogen activator inhibitor 1 (PAI-1) ELISA Kit (E-EL-M3041) were purchased from Elabscience (Wuhan, China). Fibrinogen (FIB) ELISA Kit (JL20600) was purchased from Jianglai Biotechnology (Shanghai, China). MCC950 Sodium (S7809) was purchased from Selleck (TX, USA).

### Cell culture

HEK293T and HeLa cell lines were cultured in Dulbecco’s modified Eagle’s medium (DMEM) supplemented with 10% fetal bovine serum (FBS) and 1% penicillin/streptomycin, at 37 °C and 5% CO_2_. WT and *Yod1*^*−/−*^ bone marrow-derived macrophages (BMDMs) were obtained from the mice bone marrow and grown in RPMI-1640 with 10% FBS, 1% penicillin/streptomycin and macrophage colony stimulating factor (M-CSF) (10 ng/mL) at 37 °C and 5% CO_2_ for 3 days, and the fresh medium with M-CSF was added into cell culture medium. Resident peritoneal macrophages (PMs) from C57BL/6J mice (6–8 weeks old) were harvested and cultured in RPMI-1640 medium supplemented with 10% FBS for 2 h. Then, removed the non-adherent cells and the adherent monolayer cells were used as PMs [35].

### Bacterial strains

MRSA used in this study was kindly provided by Jiandong Zhang, The Third Central Hospital of Tianjin. MRSA was identified by drug susceptibility assay in our lab. MRSA bacterial colonies were resuspended in brain heart infusion (BHI) broth and grown overnight at 37 °C with shaking. One volume of bacterial suspension was added into 100 volumes of fresh BHI broth, and incubated to logarithmic growth phase after 4 h. The bacteria were washed three times with PBS and centrifuged at 3500 r.p.m. for 15 min at 4 °C. Then, the pellet was resuspended in PBS supplemented with 10% glycerol and stored in aliquots at −80 °C. Bacterial concentration was determined by plating serial 10-fold dilution on trypsin soy agar (TSA) plates and counting colony numbers 24 h after incubation.

### Plasmid transfection

Human *YOD1* and murine *Yod1* complementary DNA (cDNA) were cloned into pcDNA3.0-Myc vector. Human *NLRP3*, *Caspase1*, and *ASC* were cloned into pcDNA3.0-Flag vector. Deleted, truncated and point mutants were obtained by PCR-based amplification and the construct encoding WT proteins as templates. Ubiquitin and its mutants (HA-Ub-WT, HA-Ub-K6, HA-Ub-K11, HA-Ub-K27, HA-Ub-K29, HA-Ub-K33, HA-Ub-K48, and HA-Ub-K63) were gifted from Xi Wang (Tiantan Hospital of Capital Medical University, Beijing, China). All constructs were confirmed by DNA sequencing. Plasmids were transiently transfected into HEK293T cells or PMs with lipofectamine 2000 according to the manufacturer’s instructions.

### ELISA

Mouse plasma concentrations of D-Dimer, TAT, FIB, PAI-1, and IL-1β were measured using commercial ELISA kits according to the manufacturer’s instructions. Briefly, blood was extracted by heart puncture from anesthetized mice by adding 3.8% sodium citrate. Plasma was isolated after centrifugation (6000 × *g*, 15 min) at 4 °C.

### Total platelet count

Blood was collected via heart puncture by adding EDTA-K2. Total platelet count was acquired by Maccura DH-510 auto hematology analyzer.

### Quantitative real-time PCR

Total RNA was extracted using TRIzol reagent and reverse-transcribed to cDNA using a commercial kit (Vazyme, Nanjing, China) according to the manufacturer’s instructions. Quantitative real-time PCR was performed with SYBR Green incorporation on Roche LightCycler­480 Instrument II, and data were normalized to *Gapdh* expression in each individual sample. The specific primers used for RT-PCR assays were listed as below: M-*Yod1*-S: 5’-GCCAAATCGCCGCTATCAC-3’; M-*Yod1*-AS: 5’-ATGTCCCGGTCGCTGAGAT-3’; M-*Gapdh*-S: 5’-AACTTTGGCATTGTGGAAGG-3’; M-*Gapdh*-AS: 5’-ACACATTGGGGGTAGGAACA-3’.

### Immunoprecipitation and immunoblot analysis

HEK293T cells post transfection or macrophages post infection, cells were lysed in IP buffer (20 mM Tris, 0.5% Triton X-100, 10% glycerol, 150 mM NaCl, 20 mM MgCl_2_, 1 mM EDTA, 1 mM PMSF pH7.5) at 4 °C for 30 min. After centrifugation at 12,000 r.p.m. for 15 min, supernatants were collected and incubated with anti-Flag M2 magnetic agarose or anti-Myc magnetic agarose or with specific antibodies and protein G plus-agarose overnight at 4 °C. Then the beads were washed three times with IP buffer. Immunoprecipitates were eluted by boiling with 1% SDS sample buffer. For immunoblot analysis, cells were lysed with RIPA buffer supplemented with a protease inhibitor “cocktail,” then the total protein concentration was measured by a bicinchoninic acid protein assay kit (Thermo Fisher Scientific, USA). Equal amounts of extracted were separated by SDS-polyacrylamide gel electrophoresis (PAGE) and then transferred to PVDF membranes for immunoblot analysis.

### Immunofluorescence staining and confocal analysis

HeLa cells were seeded onto glass coverslips in 24-well plates and cultured overnight, and then transiently transfected with plasmids encoding Myc-YOD1 and Flag-NLRP3 for 24 h. After that, the cells were washed with PBS 3 times, and fixed in 4% paraformaldehyde at room temperature for 15 min followed by permeabilization with 0.1% Triton X-100 for 10 min. Then, the cells were blocked with 5% BSA buffer at 37 °C for 1 h and incubated overnight with anti-Myc antibody and anti-Flag antibody followed by staining with Alexa Fluor 488-labeled secondary antibody and Alexa Fluor 594-conjugated secondary antibody. Nuclei were stained with DAPI (Solarbio Science and Technology, S2110). Cells were visualized using a confocal fluorescence microscope (Olympus FV1000).

### Measurement of NLRP3 inflammasome activation

WT and *Yod1*^*−/−*^ BMDMs were infected with MRSA or PBS for 4 h. To measure NLRP3 inflammasome activation, the BMDMs were lysed and performed western blot to evaluate NLRP3 expression. Furthermore, the supernatants of BMDMs were collected and concentrated to detect the expression of p20 and p17, and the production of IL-1β in supernatants was detected by ELISA.

### ASC speck formation

WT and *Yod1*^*−/−*^ BMDMs were infected with MRSA or PBS for 4 h. Then, the cells were fixed with 4% paraformaldehyde and permeabilized with 0.1% Triton X-100. ASC was stained with a secondary antibody conjugated to Alexa Fluor 488 and nuclei were stained with DAPI. The cells were visualized under fluorescence microscope (Nikon ti ECLIPSE).

### Hematoxylin and eosin (H&E) staining

Fresh tissue was fixed with 4% paraformaldehyde, embedded with paraffin, and sectioned into 5 μm thick slices. After that, the sections were stained with a H&E staining kit (Solarbio Science and Technology, G1120) in accordance with the manufacturer’s instructions. The stained slices were observed under a light microscope for morphological changes.

### MCC950 treatment

WT and *Yod1*^*−/−*^ mice were intravenously injected with 50 mg/kg (body weight) of MCC950 30 min before injection of MRSA. Mice were sacrificed at 12 h after MRSA administration. The plasma of mice was collected to analyze the levels of D-Dimer, FIB, TAT, and PAI-1 by ELISA.

### Statistical analysis

Statistical significance was calculated by performing one-way analysis of variance with Dunnett post hoc test for the comparison of three or more groups with one variable, using Prism software (GraphPad, San Diego, CA). All *p* values <0.05 were considered to be statistically significant.

## Results

### YOD1-deficient mice were susceptible to sepsis-induced DIC

YOD1 is a deubiquitinase of the ovarian tumor family. We first measured YOD1 expression in mouse PMs and BMDMs infected with MRSA. The mRNA and protein expression levels of YOD1 in both cells infected with MRSA were higher than those in uninfected macrophages (Fig. S1A–F). Furthermore, YOD1 expression was also increased in mice infected with MRSA compared to the control group (Fig. S1G–I). These data demonstrated that MRSA infection caused up-regulation of YOD1.

**Fig. 1:**
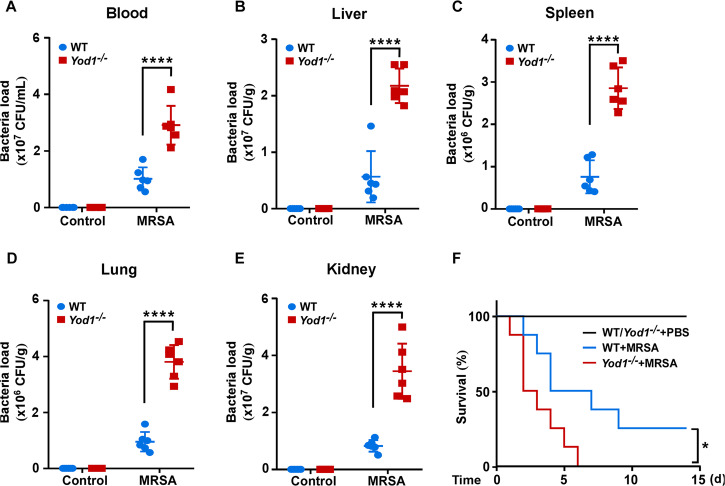
YOD1 was essential for preventing MRSA-induced septic death. WT and *Yod1*^−/−^ mice were injected intravenously with PBS (Control) or MRSA (1 × 10^8^ CFU/mouse). **A**–**E** The content of MRSA in the blood and major organs (liver, spleen, lung and kidney) of mice was analyzed at 12 h after MRSA injection. **F** The 14-day survival rate was observed (*n* = 8). Data are presented as mean ± SD. *p < 0.05, ****p < 0.0001.

**Fig. 2:**
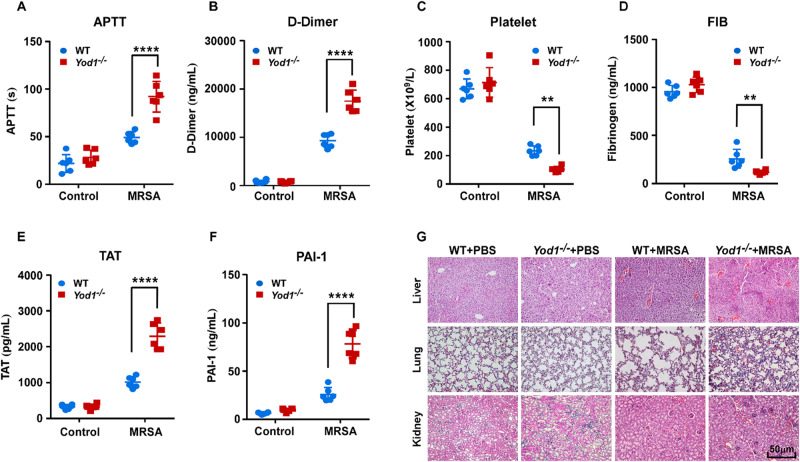
YOD1 mediated DIC in MRSA-induced sepsis. WT and *Yod1*^−/−^ mice were injected intravenously with PBS (Control) or MRSA (1 × 10^8^ CFU/mouse). Blood and tissue were collected at 12 h after MRSA injection. The levels of blood markers of DIC (APTT (**A**), D-Dimer (**B**), platelet (**C**), FIB (**D**), TAT (**E**) and PAI-1 (**F**)) were assayed. **G** Representative images of H&E staining of the major organs (liver, lung, and kidney) from mice in each group. Scale bar, 50 μm. Data are presented as mean ± SD. **p < 0.01, ****p < 0.0001.

To explore the potential function of YOD1 in the progression of sepsis-induced DIC in vivo, we generated *Yod1*-knockout mice and constructed a sepsis mouse model by intravenous injection of MRSA. We found that the average MRSA load in blood, liver, spleen, lung, and kidney of the *Yod1*^*−/−*^ mice was obviously higher than that of WT mice, respectively (Fig. 1A–E). Furthermore, the deletion of *Yod1* in mice significantly decreased septic animal survival (Fig. 1F), which demonstrated that YOD1 protected against MRSA-induced septic death. Further, we determined whether YOD1 was required for coagulation, we performed a series of blood markers assays for DIC diagnosis in WT and *Yod1*^*−/−*^ mice, including activated partial thromboplastin time (APTT), D-Dimer, and platelet count. As shown in Fig. 2A–C, compared with MRSA-induced sepsis in WT mice, the APTT and D-Dimer were increased markedly in *Yod1*^*−/−*^ mice, while the platelets were decreased. During DIC, plasma FIB concentration is reduced due to the FIB being cleaved into fibrin by thrombin [36]. Thus, we subsequently detected plasma FIB concentration, and found that MRSA induced-FIB was markedly reduced by *Yod1* deficiency (Fig. 2D). Moreover, TAT was increased in MRSA-induced septic *Yod1*^*−/−*^ mice (Fig. 2E), indicating that YOD1 could attenuate the conversion rate of prothrombin to thrombin. *Yod1* deficiency also elevated plasma PAI-1 concentration, another circulating marker of DIC (Fig. 2F). Consistently, MRSA caused thrombus and inflammatory cell infiltration in the liver, lung, and kidney were increased in *Yod1*^*−/−*^ mice, as determined by H&E staining (Fig. 2G). Taken together, these results illustrated that YOD1 alleviated coagulation in sepsis.

### YOD1 interacted with NLRP3

**Fig. 3:**
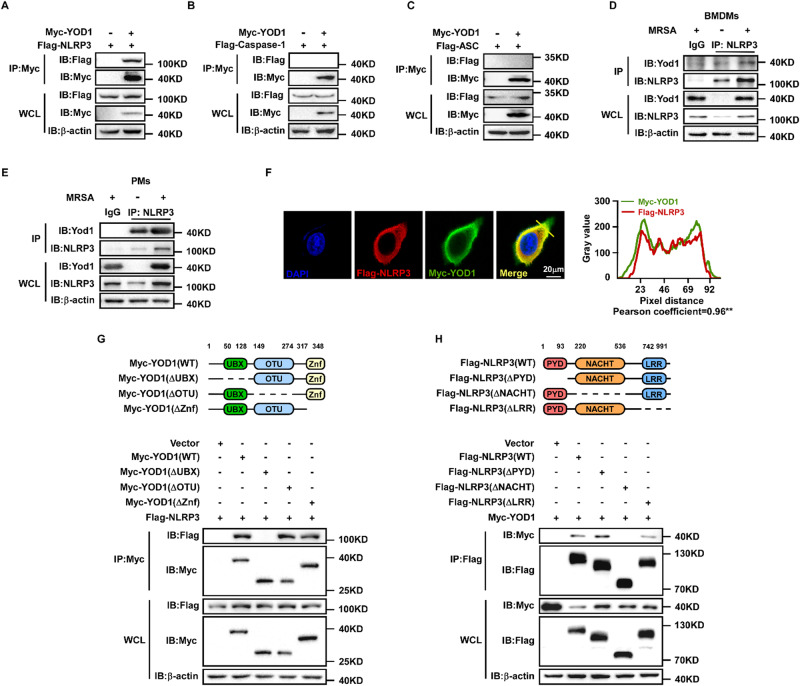
YOD1 interacted with NLRP3. **A**–**C** Immunoprecipitation and immunoblot analysis of lysates from HEK293T cells transfected with Myc-YOD1, and Flag-NLRP3 (**A**) or Flag-Caspase-1 (**B**) or Flag-ASC (**C**), followed by IP with anti-Myc, probed with anti-Flag. **D**, **E** Immunoprecipitation and immunoblot analysis of Yod1 and NLRP3 in BMDMs (**D**) or PMs (**E**) infected with MRSA for 4 h. **F** Confocal microscopy analysis of colocalization of YOD1 with NLRP3. HeLa cells transfected with Myc-YOD1 and Flag-NLRP3 for 24 h, then fixed and incubated with a secondary antibody conjugated to Alexa Fluor 488 or Alexa Fluor 594. Nuclei were labeled with DAPI. Scale bar, 20 μm. Colocalization between YOD1 and NLRP3 was analyzed by “ImageJ” software. **G** A schematic presentation of full-length YOD1 and their truncate variants (top). Myc-YOD1 or its mutants and Flag-NLRP3 were individually transfected into HEK293T cells for 24 h. Then cells were harvested and the lysates were immunoprecipitated with anti-Myc and then subjected to immunoblot analysis with the indicated Abs. **H** A schematic presentation of full-length NLRP3 and their truncate variants (top). Flag-NLRP3 or its mutants and Myc-YOD1 were individually transfected into HEK293T cells for 24 h. Then cells were harvested and the lysates were immunoprecipitated with anti-Flag and then subjected to immunoblot analysis with the indicated Abs.

To determine the molecular regulatory mechanism of YOD1, a murine Yod1 overexpression plasmid labeled with Myc was constructed and transferred into PMs to identify the targeting protein of YOD1 through immunoprecipitation and mass spectrometry (MS). We identified several YOD1-interacting proteins, including TRIM33 [37] and NEDD4 [32]. More interestingly, we also found NLRP3, a component of the inflammasome that has not previously been reported as a YOD1-interacting protein. (Fig. S2A). To verify the MS results and determine whether YOD1 interacted with other NLRP3 inflammasome molecules, we overexpressed Myc-tagged YOD1 with Flag-tagged NLRP3 or Flag-tagged Caspase-1 or Flag-tagged ASC in HEK293T cells, respectively (Figs. 3A–C and S2B–D). In vitro co-IP experiments indicated that YOD1 only interacted with NLRP3, but not with Caspase-1 and ASC. Furthermore, endogenous co-IP showed that YOD1 and NLRP3 formed a complex in both BMDMs and PMs after MRSA infection (Figs. 3D, E and S2E–H). Consistently, confocal analysis also demonstrated the colocalization between YOD1 and NLRP3 (Figs. 3F and S2I, J). These findings indicated that YOD1 specifically interacted with NLRP3.

To search for the domain of YOD1 that is required for the interaction with NLRP3, several Myc-tagged YOD1 truncated variants were constructed based on its three-subdomain including an N-terminal UBX domain, a central otubain (OTU) domain, and a C-terminal Zinc finger (Znf) domain. NLRP3 was coprecipitated with full-length YOD1, OTU domain deletion mutant and Znf domain deletion mutant, but not with UBX domain mutant (Fig. 3G). These data indicated that the UBX domain of YOD1 was required for its binding to NLRP3. NLRP3 is composed of a PYD domain, a NACHT domain, and an LRR domain. We next investigated which domain of NLRP3 is responsible for interaction with YOD1. Co-IP experiments showed that deletion of the NACHT or LRR domains dramatically reduced or obviously abolished its interaction with YOD1, while deletion of the PYD domain had little effect on YOD1 interaction (Fig. 3H). Taken together, these results showed that NLRP3 interacted with YOD1 through its NACHT and LRR domains.

### YOD1 attenuated NLRP3 expression and inflammation activation in vitro and in vivo

**Fig. 4:**
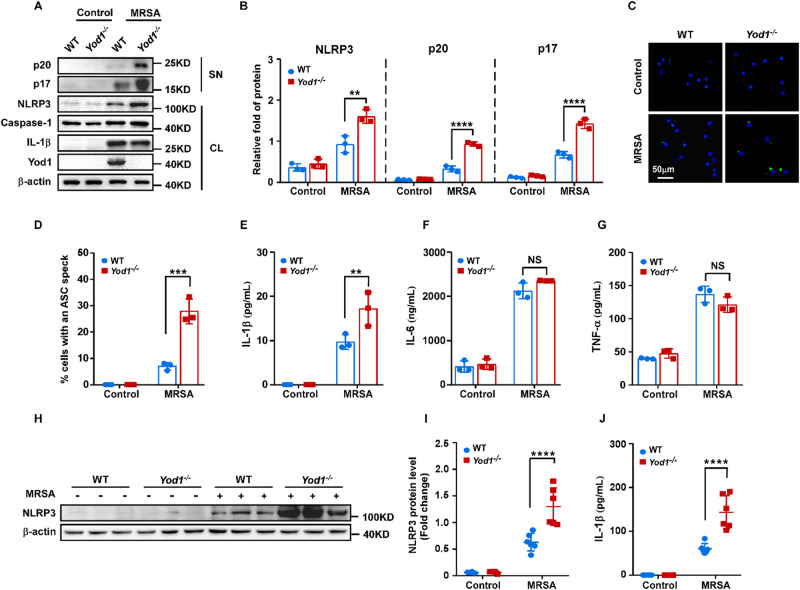
YOD1 suppressed NLRP3 inflammasome activation in vitro and in vivo. **A**, **B** Immunoblot analysis of supernatants (SN) or cell lysates (CL) of BMDMs from WT and *Yod1*^*−/−*^ mice, then treated with PBS or MRSA for 4 h. NLRP3, p20 and p17 expression levels were quantitated by measuring band intensities using “ImageJ” software. The values were normalized to β-actin. **C**, **D** Representative images of ASC specks in MRSA-infected WT and *Yod1*^*−/−*^ BMDMs. ASC, green; nuclei, blue. Scale bar, 50 μm. The percentage of cells containing an ASC speck was quantified. **E**–**G** ELISA of IL-1β (**E**), IL-6 (**F**), and TNF-α (**G**) concentration in supernatants of BMDMs from WT and *Yod1*^*−/−*^ mice after MRSA infection. **H**, **I** Immunoblot analysis of liver tissue from WT and *Yod1*^*−/−*^ mice. NLRP3 expression levels were quantitated by measuring band intensities using “ImageJ” software. The values were normalized to β-actin. **J** ELISA analysis of plasma levels of IL-1β from WT and *Yod1*^*−/−*^ mice after MRSA injection. Data are presented as mean ± SD. **p < 0.01, ***p < 0.001, ****p < 0.0001, NS means no significance.

Although YOD1 interacted with NLRP3, the functional relationship between YOD1 and NLRP3 inflammasome remains unknown. To investigate the role of YOD1 in NLRP3 inflammasome activation, BMDMs from WT and *Yod1*^*−/−*^ mice were isolated and then infected with MRSA. As shown in Fig. 4A, B, *Yod1* deficiency considerably enhanced NLRP3 expression, and Caspase-1 (p20) and IL-1β (p17) cleavage in MRSA-infected BMDMs. Activation of NLRP3 inflammasome induces oligomerization of ASCs, leading to the formation of ASC specks through the pyrin domain. As expected, the deletion of *Yod1* markedly increased the ASC speck formation (Fig. 4C, D). Meanwhile, the production of IL-1β was markedly enhanced in the absence of YOD1 (Fig. 4E). However, IL-6 and TNF-α secretion were not influenced by *Yod1* deficiency (Fig. 4F, G). Collectively, these data indicated that YOD1 suppressed NLRP3 inflammasome activation in vitro.

Next, we aimed to explore the biological effects of YOD1 on NLRP3 inflammasome activation in vivo. Compared to the WT mice, the expression of NLRP3 in liver tissue from *Yod1*^*−/−*^ mice was markedly increased (Fig. 4H, I). Furthermore, the plasma concentration of IL-1β was also enhanced significantly in *Yod1*^*−/−*^ mice (Fig. 4J). These results indicated that YOD1 could attenuate NLRP3 inflammasome activation in vivo.

### YOD1 suppressed coagulation primarily via inhibiting NLRP3 inflammasome activation

Recent studies have revealed that inflammasome activation is the major cause of systemic coagulation in lipopolysaccharide (LPS)-induced sepsis [38, 39]. We wondered whether NLRP3 inflammasome was involved in inducing coagulation in MRSA sepsis. We first investigated the effect of NLRP3 inflammasome on MRSA-induced DIC through pretreating C57BL/6J mice with MCC950, a specific inhibitor of NLRP3 inflammasome. Similarly, treatment with MCC950 prominently decreased the plasma levels of APTT, D-Dimer, TAT and PAI-1, enhanced platelet, reduced thrombus in liver, lung and kidney (Fig. S3A–F), and significantly increased septic animal survival (Fig. S3G), indicating that the NLRP3 inflammasome promoted MRSA-induced coagulation cascade.

**Fig. 5:**
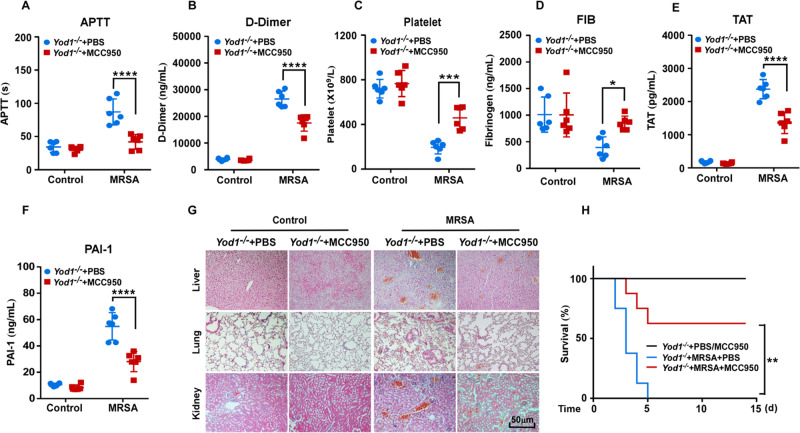
YOD1 inhibited coagulation via suppressing NLRP3 inflammasome activation. *Yod1*^*−/−*^ mice were pretreated with MCC950 for 30 min, before intravenous injection of MRSA. Mice were sacrificed at 12 h after MRSA administration. The levels of blood markers of DIC (APTT (**A**), D-Dimer (**B**), platelet (**C**), FIB (**D**), TAT (**E**), and PAI-1 (**F**)) were assayed. **G** Representative images of H&E staining of the major organs (liver, lung and kidney) from mice in each group. Scale bar, 50 μm. **H** The 14-day survival rate was observed after MCC950 treatment (*n* = 8). Data are presented as mean ± SD. *p < 0.05, **p < 0.01, ***p < 0.001, ****p < 0.0001.

Our previous results demonstrated that YOD1 specially interacted with NLRP3 and inhibited NLRP3 inflammasome activation, however, the functional relationship between YOD1, NLRP3 inflammasome and blood clotting remained unknown. We hypothesized that YOD1 suppressed coagulation primarily via inhibiting NLRP3 inflammasome activation. As expected, pretreatment with MCC950 significantly alleviated coagulation and increased animal survival caused by the deletion of the *Yod1* gene (Fig. 5A–H). Taken together, we confirmed that YOD1 suppressed coagulation primarily via inhibiting NLRP3 inflammasome activation.

### YOD1 cleaved the K33-linked ubiquitin chains on NLRP3

**Fig. 6:**
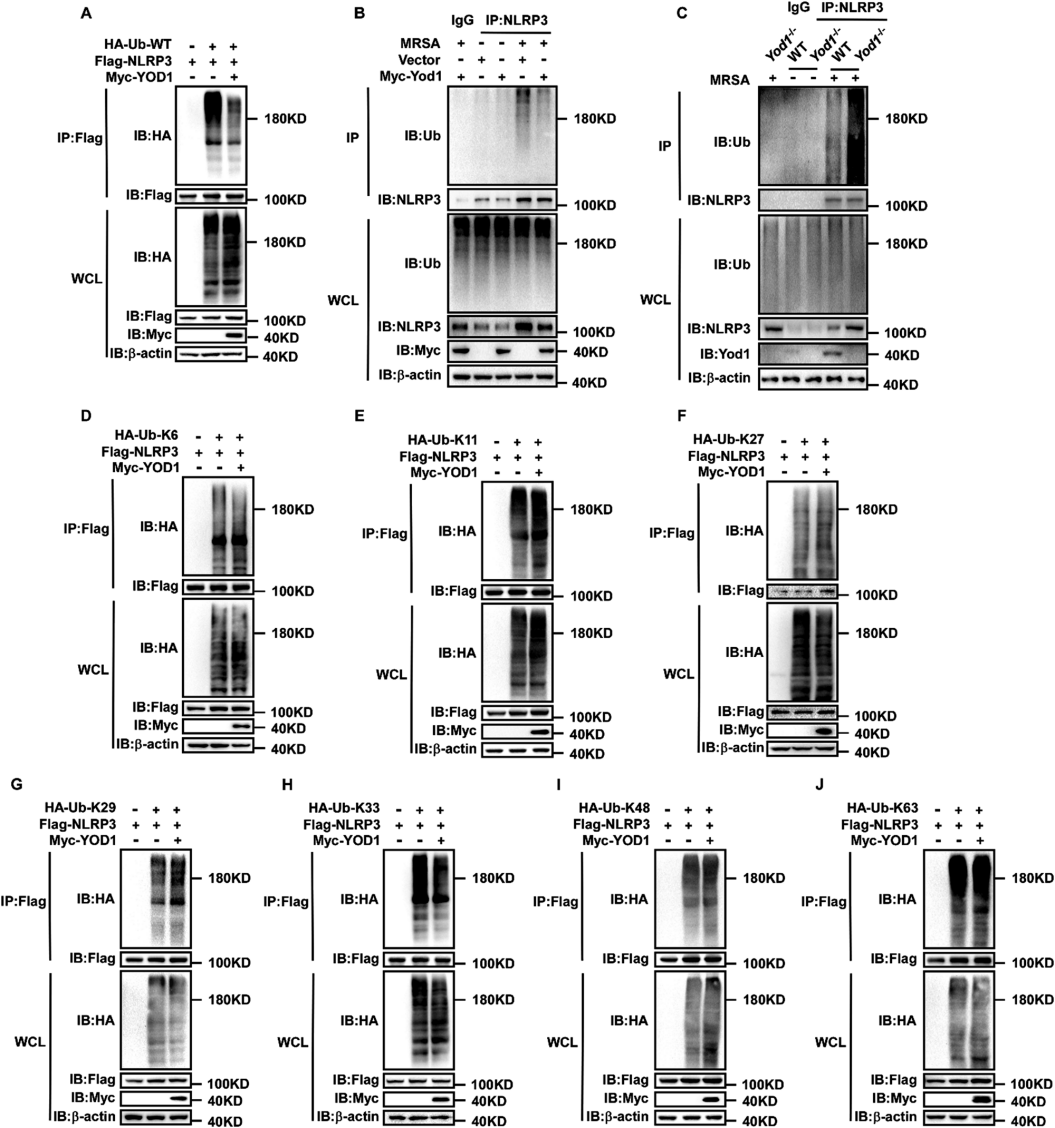
YOD1 cleaved the K33-linked ubiquitin chains on NLRP3. **A** Immunoblot analysis of lysates from HEK293T cells transfected with HA-tagged ubiquitin (HA-Ub), Flag-NLRP3 and Myc-YOD1, followed by IP with anti-Flag, probed with anti-HA. **B** Immunoblot analysis of lysates from PMs transfected with Myc-Yod1 then infected with MRSA for 4 h, followed by IP with anti-NLRP3, probed with anti-Ub. **C** Immunoblot analysis of lysates from WT and *Yod1*^*−/−*^ mice BMDMs infected with MRSA for 4 h, followed by IP with anti-NLRP3, probed with anti-Ub. **D**–**J** Immunoblot analysis of lysates from HEK293T cells transfected with HA-tagged K6-/K11-/K27-/K29-/K33-/K48-/K63-linked ubiquitin, Flag-NLRP3 and Myc-YOD1, followed by IP with anti-Flag, probed with anti-HA.

It is well established that upregulation of NLRP3 expression is critical for the activation of NLRP3 inflammasome. Intriguingly, overexpression of YOD1 greatly decreased NLRP3 protein level rather than NLRP3 mRNA expression in MRSA-infected PMs, suggesting that YOD1 regulated NLRP3 expression through post-translational modifications (Fig. S4A–C). We next determined whether YOD1 specifically mediated the ubiquitination of NLRP3. NLRP3 inflammasome components (Flag-tagged NLRP3, ASC and Caspase-1) were co-transfected with HA-tagged WT ubiquitin and Myc-tagged WT YOD1 into HEK293T cells. As shown in Figs. 6A and S4D, E, YOD1 significantly reduced the ubiquitination of NLRP3, while ASC and Caspase-1 did not, suggesting that YOD1 inhibited NLRP3 inflammasome activation by cleaving NLRP3 ubiquitination. To further delineate the role of YOD1 in regulating the ubiquitination of NLRP3, we measured the endogenous ubiquitination of NLRP3 in MRSA-infected primary macrophages by specific WT ubiquitin Ab. As shown in Fig. 6B, C, NLRP3 was robustly ubiquitinated with WT chains after MRSA infection, indicating activation of NLRP3 inflammasome. However, compared with the control group, overexpression of Yod1 abrogated WT ubiquitination of NLRP3 (Fig. 6B), while knockout of *Yod1* significantly enhanced them (Fig. 6C). NLRP3 could be modified by various types of ubiquitination, each with different biological function. To distinguish which lysine-linked ubiquitin of NLRP3 was regulated by YOD1, we used a series of ubiquitin mutants with only one indicated lysine available for linkage (K6, K11, K27, K29, K33, K48 and K63). Notably, only K33-linked ubiquitination of NLRP3 was markedly decreased, while other types of ubiquitination were not affected when YOD1 was overexpressed under the same conditions (Fig. 6D–J). Collectively, these data demonstrated that YOD1 regulated NLRP3 inflammasome activation via cleaving K33-linked ubiquitination chains.

### YOD1 regulated NLRP3 dependent on its enzymatic activity

**Fig. 7:**
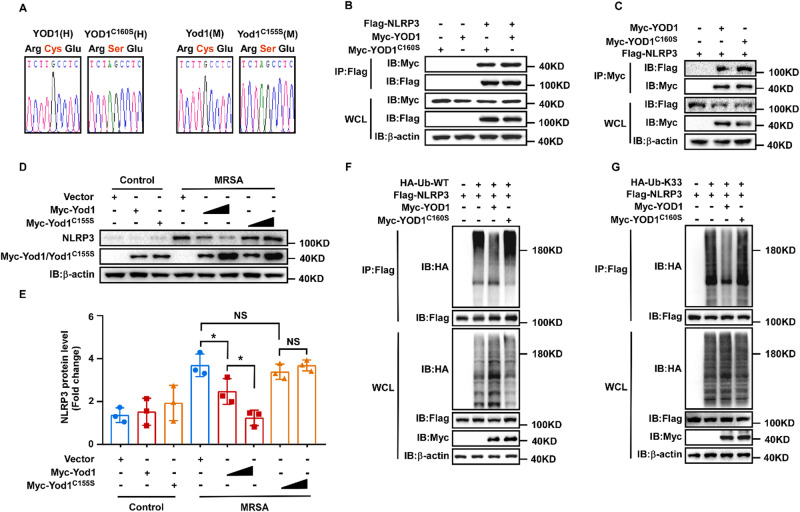
YOD1 regulated NLRP3 dependent on its enzymatic activity. **A** DNA sequencing result of plasmids expressing human Myc-YOD1 and YOD1^C160S^ or mouse Myc-Yod1 and Yod1^C155S^. **B**, **C** Immunoprecipitation and immunoblot analysis of the interaction between Flag-NLRP3 and Myc-YOD1 or Myc-YOD1^C160S^ in HEK293T cells. Flag-NLRP3 and Myc-YOD1 or its mutants were individually transfected into HEK293T cells for 24 h. Then cells were harvested and the lysates were immunoprecipitated with anti-Flag (**B**) or anti-Myc (**C**) and then subjected to immunoblot analysis with the indicated Abs. **D**, **E** Immunoblot analysis of PMs transfected with Myc-Yod1 or Myc-Yod1^C155S^ then infected with MRSA for 4 h. NLRP3 expression level was quantitated by measuring band intensities using “ImageJ” software. The values were normalized to β-actin. **F** Immunoblot analysis of lysates from HEK293T cells transfected with HA-Ub, Flag-NLRP3, Myc-YOD1 and Myc-YOD1^C160S^, followed by IP with anti-Flag, probed with anti-HA. **G** Immunoblot analysis of lysates from HEK293T cells transfected with HA-K33-Ub, Flag-NLRP3, Myc-YOD1 and Myc-YOD1^C160S^, followed by IP with anti-Flag, probed with anti-HA. Data are presented as mean ± SD. *p < 0.05, NS means no significance.

YOD1, a deubiquitinase, has been reported to regulate MAVS activation and aggregation through its catalytical activity [33]. Given that YOD1 specifically cleaved K33-linked ubiquitination of NLRP3, we thought that YOD1 suppressed inflammasome activation by virtue of its enzymatic activity. To test our hypothesis, we first constructed human and mouse YOD1 point mutations respectively, a catalytically inactive version (Fig. 7A). As expected, the interaction between YOD1 and NLRP3 remained robust after mutation (Fig. 7B, C). However, the protein level of NLRP3 induced by MRSA decreased in a dosage-dependent after Yod1 was transfected into macrophages, whereas Yod1(C155S) did not affect NLRP3 expression (Fig. 7D, E), indicating that YOD1 suppressed NLRP3 expression through its enzymatic activity. Consistently, we also observed that the catalytic inactive mutant YOD1(C160S) lost its capacity to mediate the ubiquitination status of NLRP3 (Fig. 7F, G). Taken together, these findings indicated that YOD1 removed the K33-linked ubiquitination of NLRP3 and inhibited the activation of NLRP3 inflammasome dependent on its enzymatic activity.

## Discussion

**Fig. 8:**
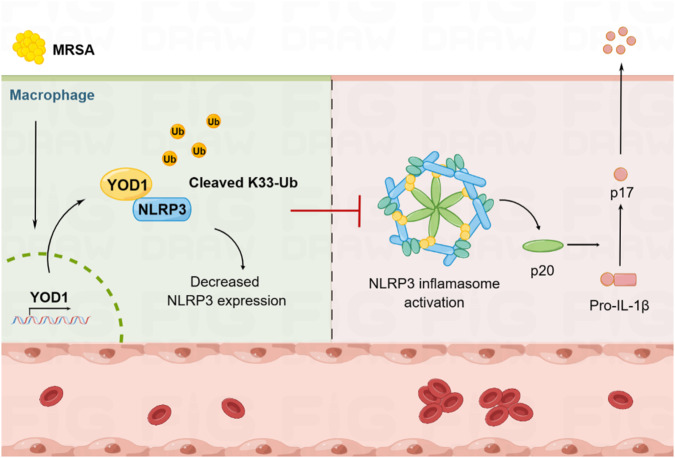
The model of YOD1 in NLRP3 inflammasome-mediated MRSA sepsis-induced DIC. MRSA induced YOD1 expression and interacted with NLRP3, cleaved K33-linked ubiquitination of NLRP3, and thus inhibited NLRP3 expression and inflammasome activation, thereby attenuating NLRP3 inflammasome-mediated coagulation. This figure was drawn with Figdraw.

NLRP3 inflammasome activation and ubiquitination are two major host innate immune events, involving multiple aspects, such as bacterial infection, autoinflammatory and autoimmune diseases [40–43]. The activity and availability of NLRP3 are stringently controlled by ubiquitination to avoid uncontrolled excessive immune responses [22]. Here, we identified the deubiquitinase YOD1 for the first time served as a specific regulator of NLRP3 inflammasome-mediated coagulation and explored the underlying mechanism (Fig. 8). *Yod1* deficiency specifically promoted NLRP3 inflammasome assembly and activation both in vitro and in vivo. Mechanistically, YOD1 was directly bound to NLRP3 through its UBX domain, the latter inhibited expression of NLRP3 through cleaving K33-linked ubiquitin chains of NLRP3. Thus, the activation of NLRP3 inflammasome was limited in a proper intensity and time course to avoid coagulation.

Our study highlighted the impact of YOD1 on the regulation of NLRP3 inflammasome-mediated coagulation activation. YOD1 is a deubiquitinase of the OTU (otubain) protein family, which has been implicated in the occurrence and progression of viral infectious diseases and a wide spectrum of cancer through removing ubiquitin chains and regulating protein degradation [31, 44–46]. We previously reported that YOD1 expression was enhanced after Sendai virus infection, upregulation of YOD1 in cells played a negative role in antiviral innate immunity. In the first place, the present study revealed that YOD1 was upregulated in MRSA-infected macrophages. Notably, we found that the number of MRSA in their blood and major organs from mice with *Yod1* gene deletion was much higher than those in WT mice with sepsis. To the best of our knowledge, the functions of YOD1 in sepsis-induced DIC have not been investigated. Our results showed that during MRSA infection, *Yod1*^*−/−*^ mice had increased APTT, D-Dimer, TAT and PAI-1, and decreased FIB and platelet count compared with control group, suggesting that YOD1 could protect the host against MRSA infection and might be a therapeutic target for DIC.

It has been an important issue for identifying the specific target protein of YOD1. NLRP3 is a member of the NLR family, which recruits ASC and Caspase-1 to form NLRP3 inflammasome. Using MS, co-IP and co-immunofluorescence data, we identified NLRP3 as a putative target protein and YOD1 specifically interacted with NLRP3, but not with ASC and Caspase-1. Furthermore, the loss of *Yod1* expression enhanced the expression of NLRP3 in vitro and in vivo. Conversely, overexpression of YOD1 decreased the expression of NLRP3 in MRSA-infected PMs. Domain mapping studies indicated that the UBX domain of YOD1 was required for its interaction with NLRP3, and the NACHT and LRR domains of NLRP3 were required for its interaction with YOD1. YOD1 contains three domains, including a UBX domain, an OTU domain and a Znf domain. It has been reported that the OTU catalytic core is necessary and sufficient for YOD1 deubiquitination activity in vitro, while the UBX and Znf domains put OTU in the appropriate place [30]. Similarly, the present study confirmed the hypothesis that the UBX domain of YOD1 caught the NACHT and LRR domains of NLRP3 and placed the OTU domain in the appropriate space to regulate the ubiquitination of NLRP3.

Inflammasomes contain a sensor, an adapter (ASC) and Caspase-1. Generally, inflammasomes can be divided into canonical inflammasome and noncanonical inflammasome, according to activation of CASP1 or CASP11 (CASP11 in mice, CASP4 and CASP5 in humans) [47, 48]. Canonical inflammasome can be further classified into NLRP3, NLRP1b, NLRC4, AIM2 and Pyrin inflammasomes [49]. NLRP3 inflammasome has been reported to be associated with a variety of diseases, such as sepsis, gout, Alzheimer’s disease, atherosclerosis, diabetes and the new COVID-19 [50–54]. Sepsis is a complex process that involves severe inflammation and organ dysfunction. DIC is a common complication of sepsis and various mechanisms may contribute to its development. Recently, the molecular mechanism of coagulation in sepsis has been revealed in studies of inflammasome activation [38, 39]. Specifically, canonical inflammasome or noncanonical inflammasome-mediated macrophage pyroptosis regulated tissue factor release, leading to blood coagulation and organ damage in sepsis. Intriguingly, the deletion of *Nlrp3* alleviated endotoxin-induced coagulation, supporting that NLRP3 inflammasome is at least one of the major mechanisms of sepsis-induced DIC [17]. In our study, we showed that YOD1 interacted with NLRP3 and suppressed the NLRP3 inflammasome activation. In addition, YOD1-mediated coagulation in MRSA-induced sepsis was blocked by the administration of MCC950, a highly potent and specific inhibitor of NLRP3, which supported that YOD1-regulated NLRP3 inflammasome-dependent coagulation in the process of MRSA-induced DIC.

In general, the deubiquitination of NLRP3 promotes its activation. Deubiquitinase BRCC3 enhances NLRP3 activation via cleaving the ubiquitin chains of NLRP3 [26]. UAF1/USP1, a deubiquitinase complex, maintains NLRP3 stability by removing its K48-linked ubiquitin [28]. USP7 and USP47 also promote NLRP3 activation [55]. Notably, YOD1 attenuated NLRP3 inflammasome activation by removing polyubiquitination chains of NLRP3, indicating that the deubiquitinase had different regulatory effect on NLRP3. It was known that K63-linked ubiquitination is the signal to maintain protein stability [56], our data provided compelling evidence that K33-linked ubiquitination might also lead to the stability of the target proteins. Moreover, our data showed that YOD1 was a special deubiquitinase for K33-, but not K6-/K11-/K27-/K29-/K48-/K63-linked ubiquitination of NLRP3. Overexpression of YOD1 decreased endogenous WT ubiquitin of NLRP3, and *Yod1* deficiency potentiated endogenous WT ubiquitin of NLRP3. Cys160 of YOD1 is the key site for its deubiquitinase activity. Although the mutation of YOD1 could interact with NLRP3, we found that the effect of YOD1 on NLRP3 ubiquitination was dependent on the deubiquitinase catalytic activity. Cells expressing YOD1 (C160S) lost the ability to remove the WT and K33-linked ubiquitination of NLRP3. Furthermore, ectopic expression of Yod1 in PMs inhibited the expression of NLRP3, while the mutant did not. These results demonstrated that YOD1 negatively regulated the NLRP3 inflammasome activation in a deubiquitinase activity-dependent manner and explored a novel function of YOD1.

In summary, our study revealed one of the essential and original mechanisms by which YOD1 promoted sepsis-induced DIC, and proposed YOD1 as a priming therapeutic target for NLRP3-inflammasome dysregulation-mediated disorders.

## Supplementary information

Supplementary Material

Original Data File

## Data Availability

All data generated or analyzed during this study are included in this published article and its Supplementary Information files.
