# Correction: YOD1 protects against MRSA sepsis-induced DIC through Lys33-linked deubiquitination of NLRP3

**DOI:** 10.1038/s41419-024-06991-1

**Published:** 2024-09-04

**Authors:** Chang Liu, Caihong Fan, Jia Liu, Shiqi Zhang, Huixin Tang, Yashan Liu, Shengzheng Zhang, Qiang Wu, Jiandong Zhang, Zhi Qi, Yanna Shen

**Affiliations:** 1 School of Medical Technology, Tianjin Medical University, Tianjin, China; 2 Key Laboratory of Emergency and Trauma of Ministry of Education, Hainan Medical University, Haikou, China; 3 Department of Molecular Pharmacology, School of Medicine, Nankai University, Tianjin, China; 4 The Third Central Hospital of Tianjin, Tianjin, China; 5 Institute of Digestive Disease, Shengli Oilfield Central Hospital, Dongying, China; 6 Tianjin Key Laboratory of General Surgery in Construction, Tianjin Union Medical Center, Tianjin, China; 7 The First Department of Critical Care Medicine, The First Affiliated Hospital of Shihezi University, Shihezi, China

**Keywords:** Ubiquitylation, Infection

Correction to: *Cell Death and Disease* 10.1038/s41419-024-06731-5, published online 24 May 2024

To accurately reflect the authors’ contributions and maintain the integrity of the publication, the sequence of authors has been updated. It reads:

^9^These authors contributed equally: Chang Liu, Caihong Fan, Jia Liu

But should read:

^8^These authors contributed equally: Chang Liu, Caihong Fan, Jia Liu.

The original article has been corrected.

