# Supplementary material for: YOD1 protects against MRSA sepsis-induced DIC through Lys33-linked deubiquitination of NLRP3
Source: Cell Death Dis. 2024 May 24;15(5):360. doi: 10.1038/s41419-024-06731-5 (PMC11126606; doi:10.1038/s41419-024-06731-5)
Supplement: Supplementary file 1 — Supplementary Material [file 41419_2024_6731_MOESM1_ESM.pdf]

Supplementary Material

YOD1 protects against MRSA sepsis-induced DIC through Lys33-linked deubiquitination of NLRP3

Chang Liu [1,3,8], Caihong Fan [1,8], Jia Liu [1,8], Shiqi Zhang [1], Huixin Tang [1], Yashan Liu [1], Shengzheng Zhang [4], Qiang Wu [3], Jiandong Zhang [7], Zhi Qi [2,4,5,6] ✉ and Yanna Shen [1] ✉

[1] School of Medical Technology, Tianjin Medical University, Tianjin, China

[2] Institute of Digestive Disease, Shengli Oilfield Central Hospital, Dongying, China

[3] Key Laboratory of Emergency and Trauma of Ministry of Education, Hainan Medical University, Haikou, China

[4] Department of Molecular Pharmacology, School of Medicine, Nankai University, Tianjin, China

[5] Tianjin Key Laboratory of General Surgery in Construction, Tianjin Union Medical Center, Tianjin, China

[6] The First Department of Critical Care Medicine, The First Affiliated Hospital of Shihezi University, Shihezi, China

[7] The Third Central Hospital of Tianjin, 83 Jintang Road, Hedong District, Tianjin, China

[8] These authors contributed equally: Chang Liu, Caihong Fan, Jia Liu

Corresponding authors: qizhi@nankai.edu.cn (Zhi Qi) ; shenyanna@tmu.edu.cn (Yanna Shen)

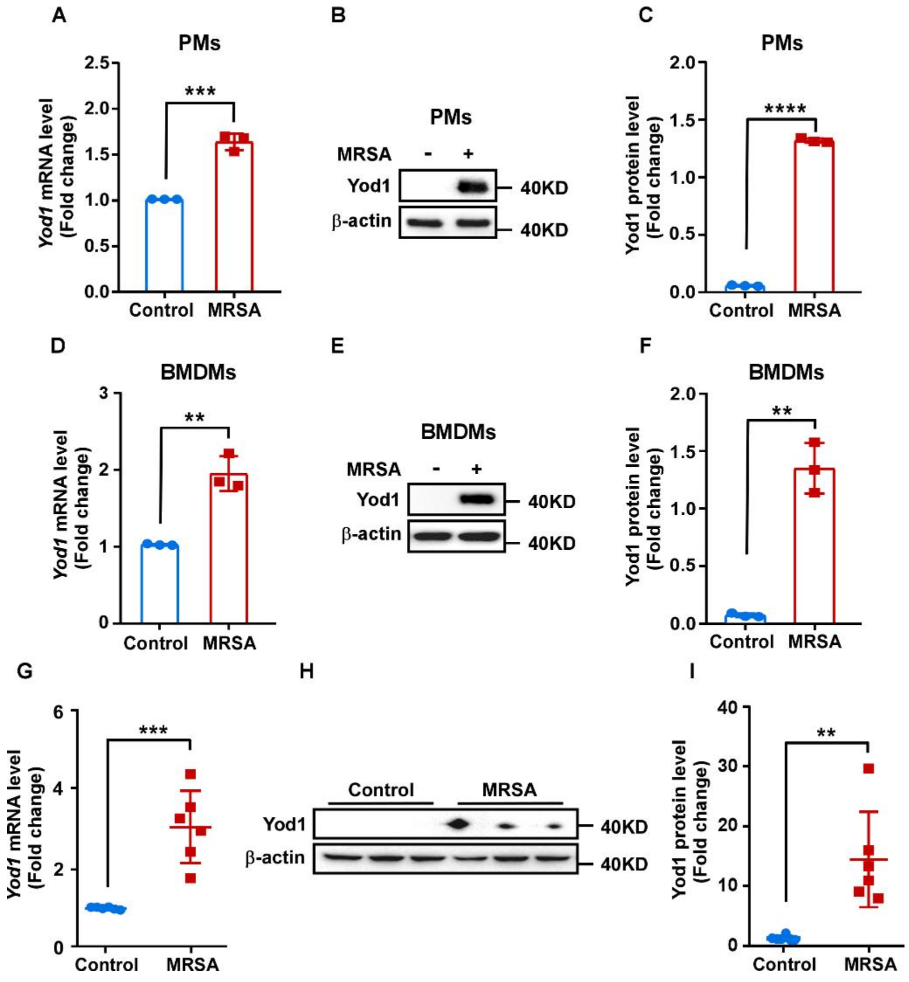

Fig S1. MRSA infection induced YOD1 expression. (A) RT-PCR analysis of *Yod1* mRNA expression in PMs upon infection with MRSA for 4 h. *Gapdh* was used as loading control. (B-C) Immunoblot analysis of Yod1 protein expression in PMs upon infection with MRSA for 4 h. Yod1 expression levels were quantitated by measuring band intensities using "ImageJ" software. The values were normalized to β-actin. (D) RT-PCR analysis of *Yod1* mRNA expression in BMDMs upon infection with MRSA for 4 h. *Gapdh* was used as loading control. (E-F) Immunoblot analysis of Yod1 protein expression in BMDMs upon infection with MRSA for 4 h. Yod1 expression levels were quantitated by measuring band intensities using "ImageJ" software. The values were normalized to β-actin. (G-I) C57BL/6J mice were injected intravenously with PBS (Control) or MRSA (1×10^8 CFU/mouse). Liver tissue was collected at 12 h after MRSA injection. (G) RT-PCR analysis of *Yod1* mRNA expression and *Gapdh* was used as loading control. (H-I) Immunoblot analysis of Yod1 protein expression. Yod1 expression levels were quantitated by measuring band intensities using

"ImageJ" software. The values were normalized to β-actin. Data are presented as mean ± SD. $^{**}p < 0.01$, $^{***}p < 0.001$, $^{****}p < 0.0001$.

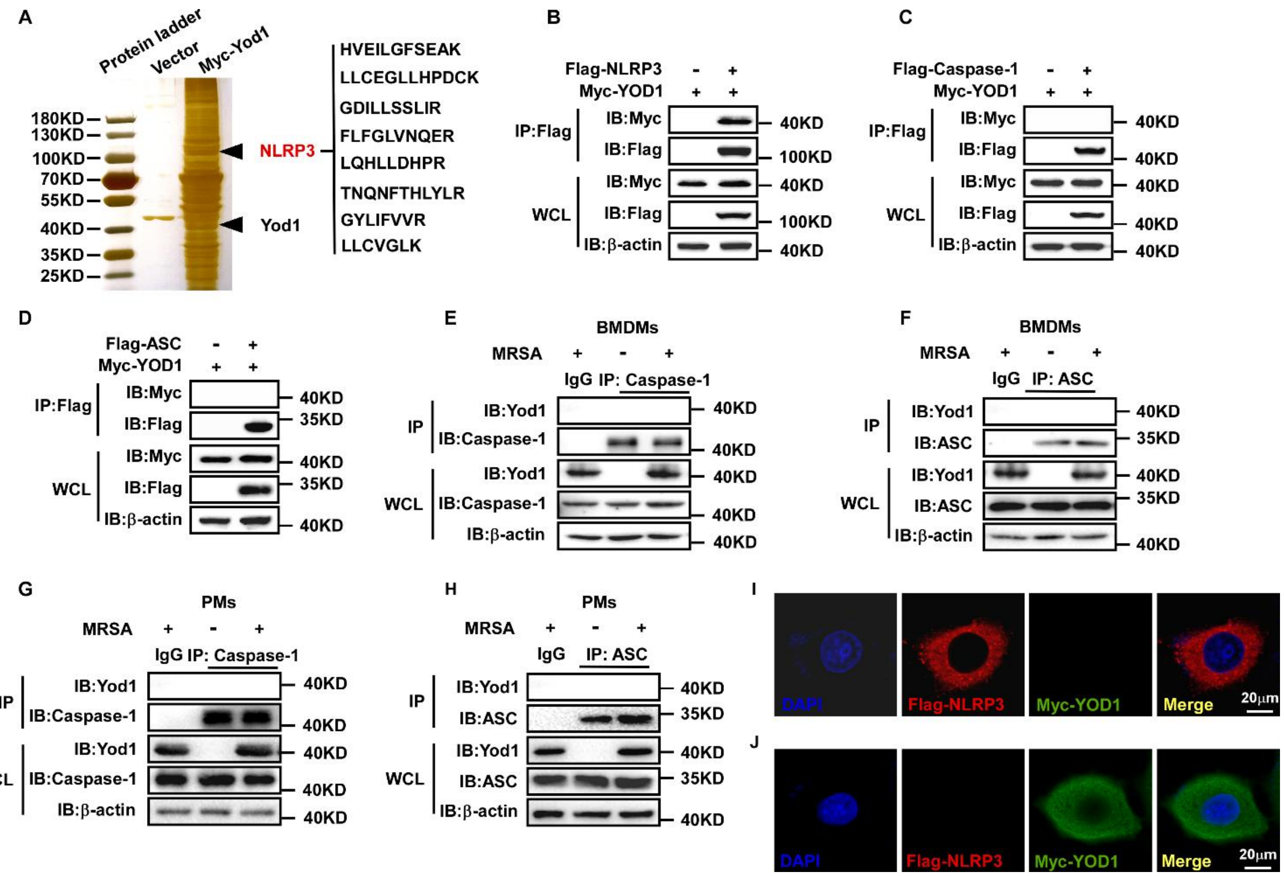

Fig S2. YOD1 interacted with NLRP3. (A) MS analysis of interaction between Yod1 and NLRP3. PMs transfected with Myc-Yod1 and infected with MRSA for 4 h, followed by IP with anti-Myc. (B-D) Immunoprecipitation and immunoblot analysis of lysates from HEK293T cells transfected with Myc-YOD1, and Flag-NLRP3 (B) or Flag-Caspase-1 (C) or Flag-ASC (D), followed by IP with anti-Flag, probed with anti-Myc. (E-F) Immunoprecipitation and immunoblot analysis of Yod1 and Caspase-1 (E) or Yod1 and ASC (F) in BMDMs infected with MRSA for 4 h. (G-H) Immunoprecipitation and immunoblot analysis of Yod1 and Caspase-1 (G) or Yod1 and ASC (H) in PMs infected with MRSA for 4 h. (I) Confocal microscopy analysis of localization of NLRP3. HeLa cells were transfected with Flag-NLRP3 for 24 h, then fixed and incubated with a secondary antibody conjugated to Alexa Fluro 594. Nuclei were labeled with DAPI. Scale bar, 20 μm. (J) Confocal microscopy analysis of localization of YOD1. HeLa cells transfected with Myc-YOD1 for 24 h, then

fixed and incubated with a secondary antibody conjugated to Alexa Fluro 488. Nuclei were labeled with DAPI. Scale bar, 20 μm.

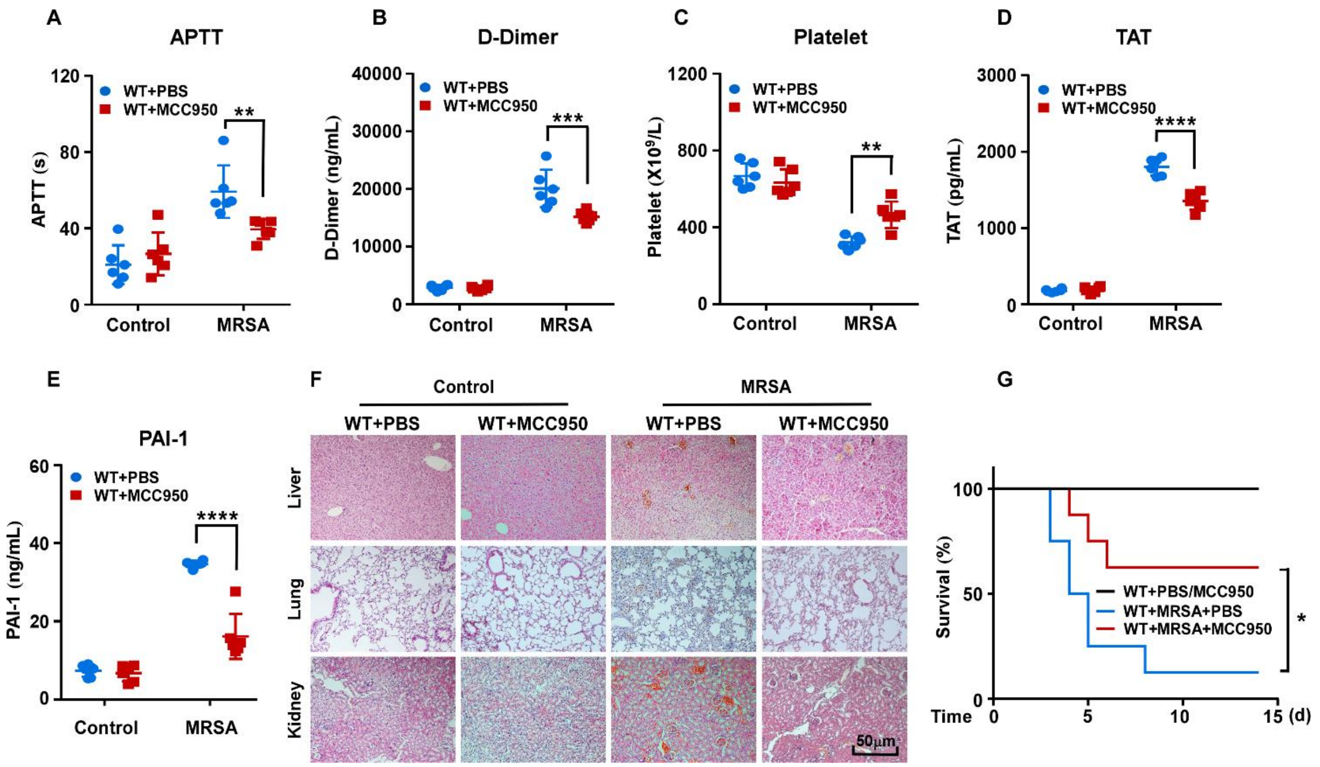

Fig S3. NLRP3 inflammasome mediated MRSA-induced activation of coagulation cascades. MRSA was injected intravenously into C57BL/6J mice pretreated with MCC950 for 30 min. Mice were sacrificed at 12 h after MRSA administration. The levels of blood markers of DIC (APTT (A), D-Dimer (B), platelet (C), TAT (D) and PAI-1(E)) were assayed. (F) Representative images of H&E staining of the major organs (liver, lung and kidney) from mice in each group. Scale bar, 50 μm. (G) The 14-day survival rate was observed after MCC950 treatment (n=8). Data are presented as mean ± SD. *$p < 0.05$, **$p < 0.01$, ***$p < 0.001$, ****$p < 0.0001$.

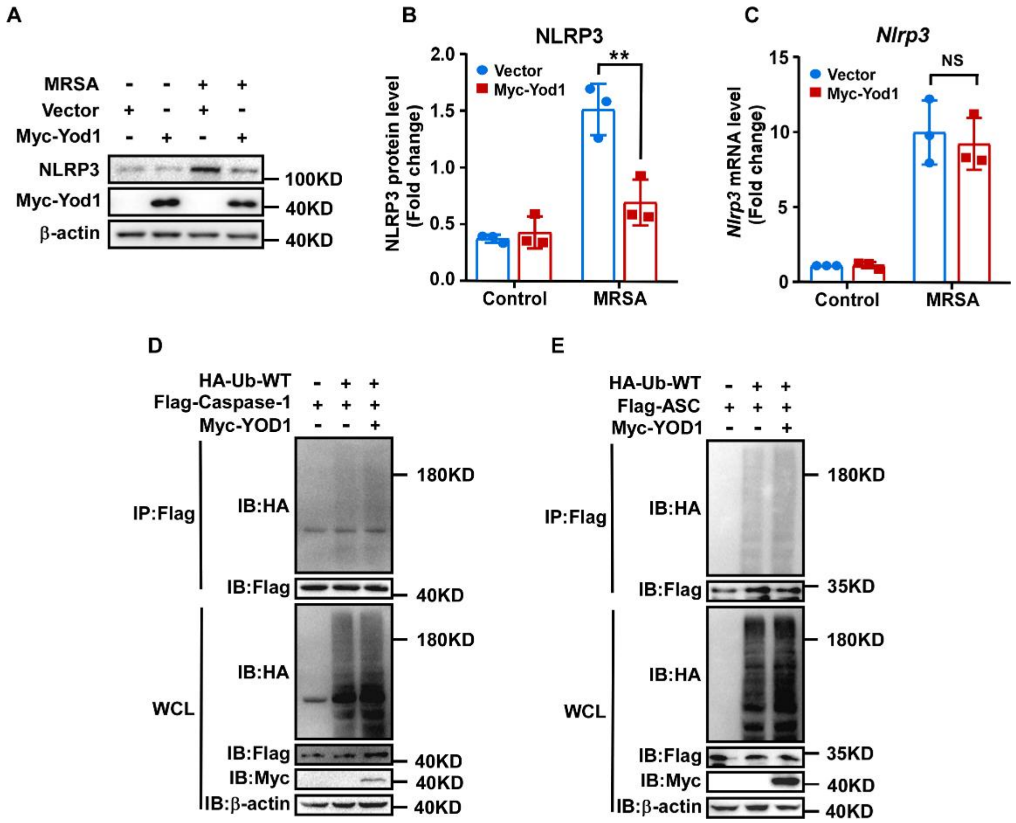

Fig S4. Overexpression of YOD1 inhibited NLRP3 expression and had no effect on Caspase-1 and ASC ubiquitination. (A-C) PMs were transfected with Myc-Yod1 for 24 h, then infected with MRSA for 4 h. (A-B) Immunoblot and quantification of NLRP3 were determined. The values were normalized to β-actin. (C) The mRNA level of *Nlrp3* gene was examined by RT-PCR. *Gapdh* was used as loading control. (D) Immunoblot analysis of lysates from HEK293T cells transfected with HA-Ub, Flag-Caspase-1 and Myc-YOD1, followed by IP with anti-Flag, probed with anti-HA. (E) Immunoblot analysis of lysates from HEK293T cells transfected with HA-Ub, Flag-ASC and Myc-YOD1, followed by IP with anti-Flag, probed with anti-HA. Data are presented as mean ± SD. $^{**}p < 0.01$, NS means no significance.
