# Supplementary material for: YOD1 protects against MRSA sepsis-induced DIC through Lys33-linked deubiquitination of NLRP3
Source: Cell Death Dis. 2024 May 24;15(5):360. doi: 10.1038/s41419-024-06731-5 (PMC11126606; doi:10.1038/s41419-024-06731-5)

Figure 3 A

1. IP：Flag-NLRP3

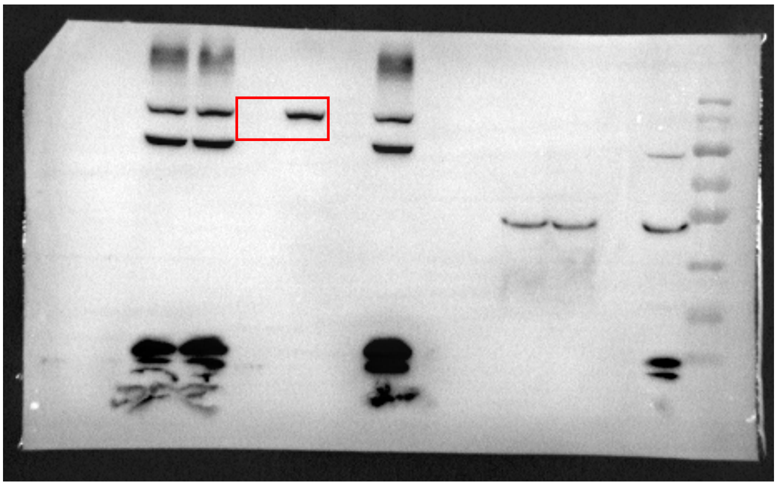

2. IP: Myc-YOD1

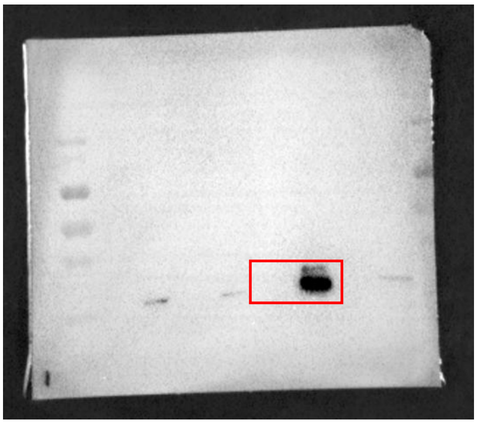

3. Input: Flag-NLRP3

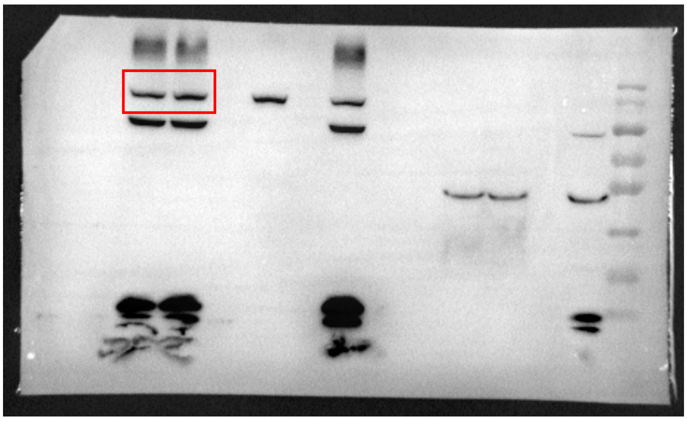

4. Input: Myc-YOD1

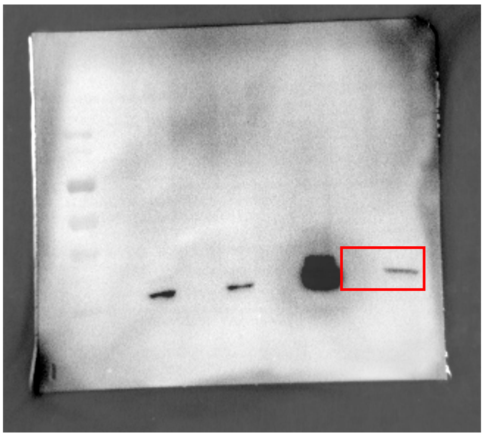

5. Actin

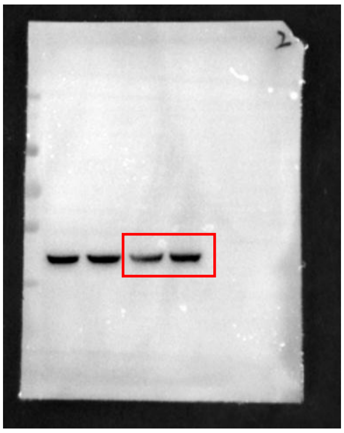

Figure 3 B

1. IP：Flag-Caspase-1

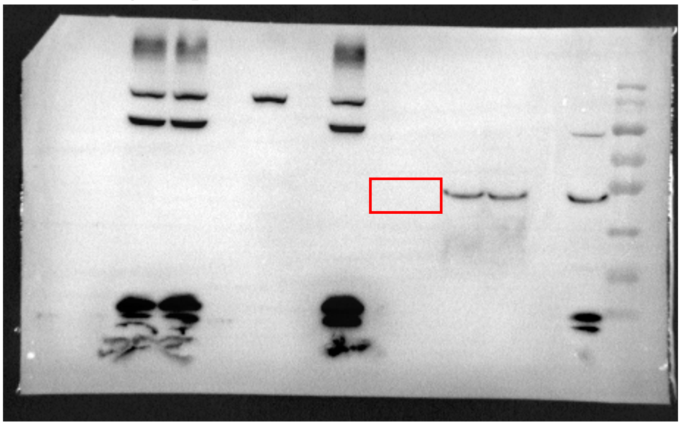

2. IP: Myc-YOD1

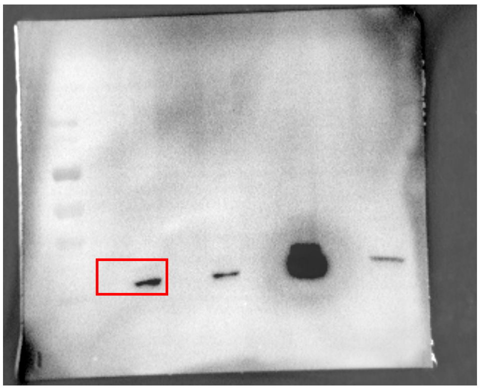

3. Input: Flag-Caspase-1

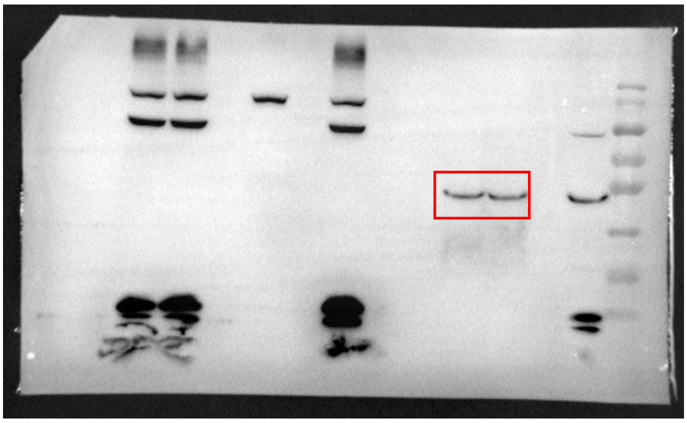

4. Input: Myc-YOD1

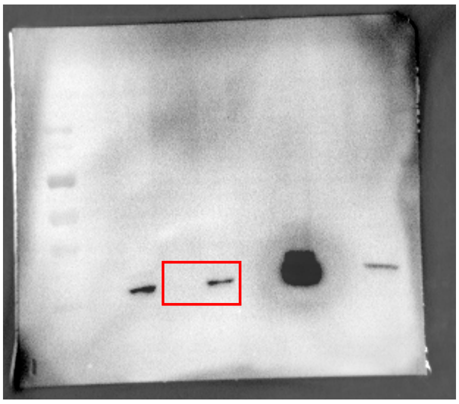

5. Actin

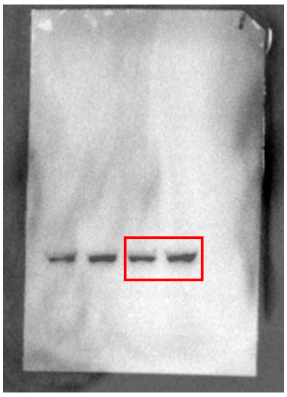

Figure 3 C

1. IP：Flag-ASC

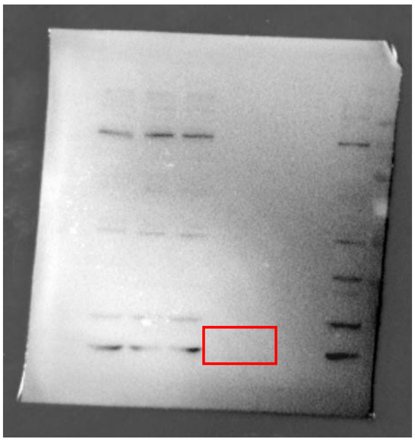

2. IP: Myc-YOD1

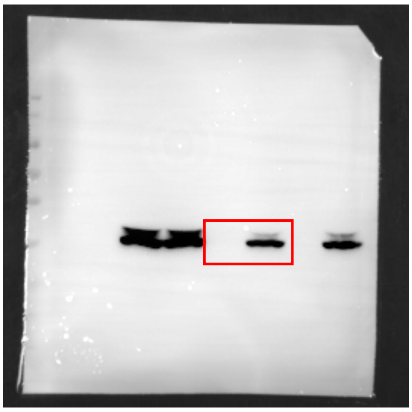

3. Input: Flag-ASC

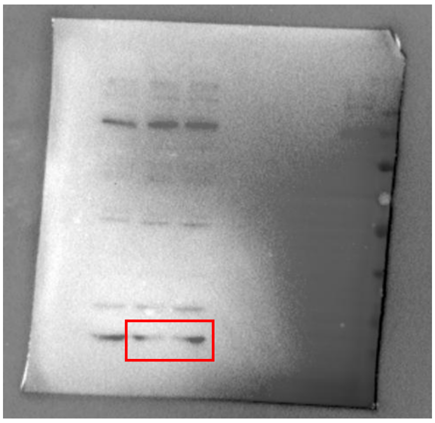

4. Input: Myc-YOD1

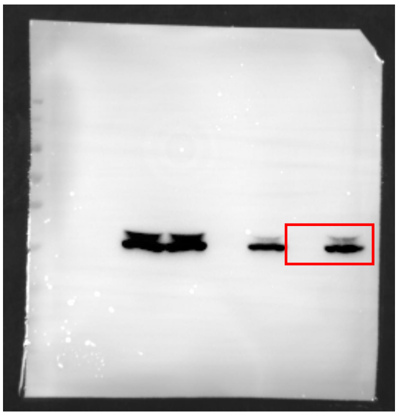

5. Actin

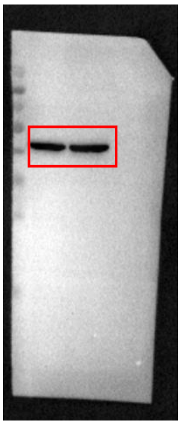

Figure 3 D

1. IP: YOD1

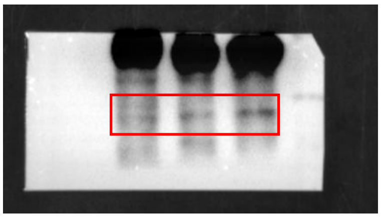

2. IP: NLRP3

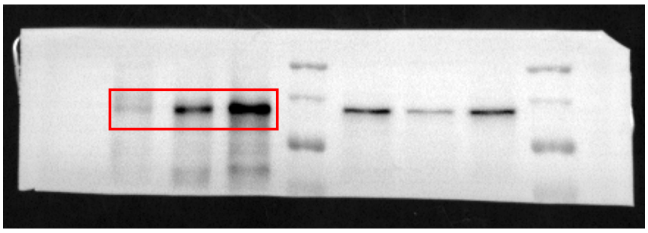

3. Input: YOD1

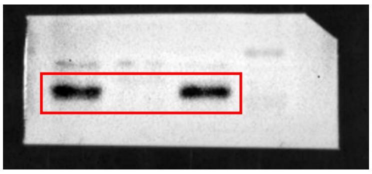

4. Input: NLRP3

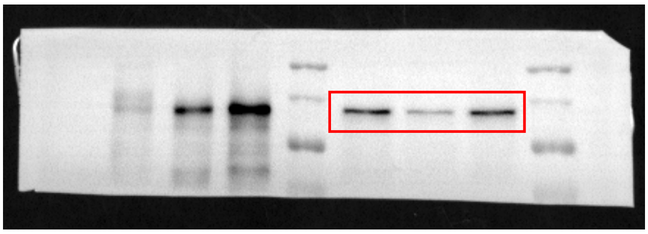

5. Actin

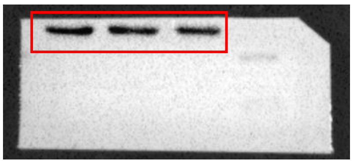

Figure 3 E

1. IP: YOD1

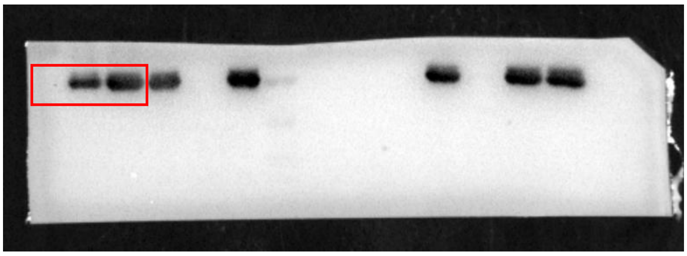

2. IP: NLRP3

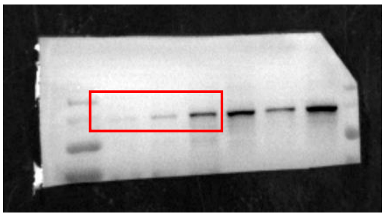

3. Input: YOD1

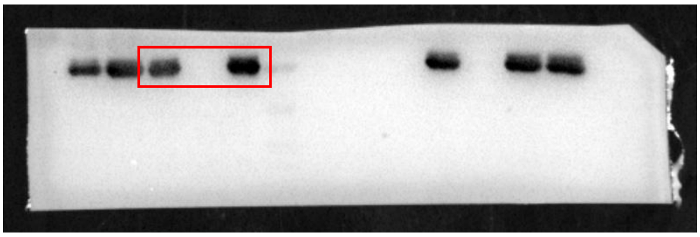

4. Input: NLRP3

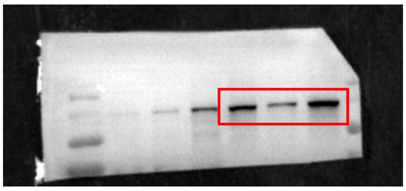

5. Actin

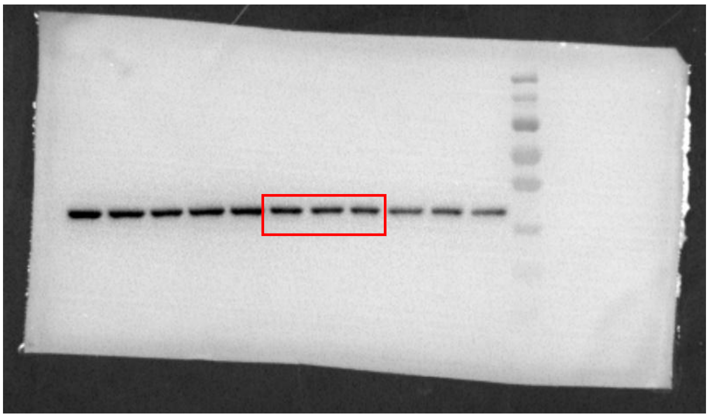

Figure 3 G

1. IP: Flag-NLRP3

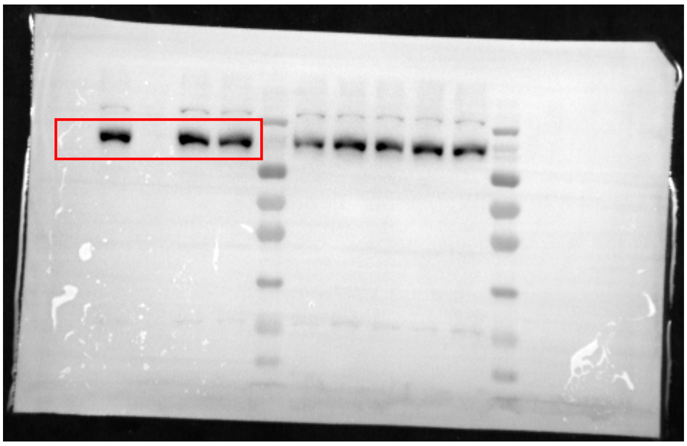

2. IP: Myc-YOD1

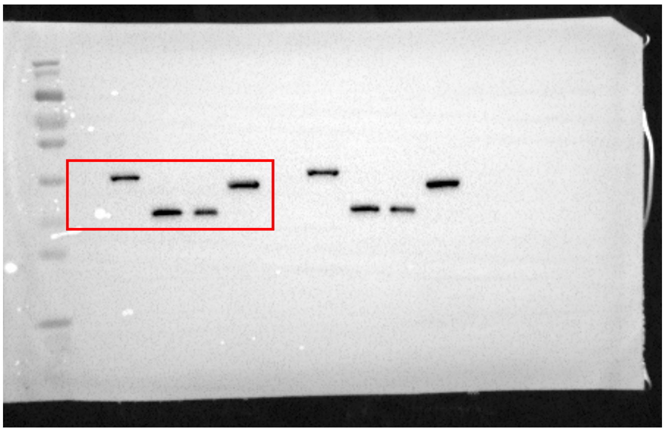

3. Input: Flag-NLRP3

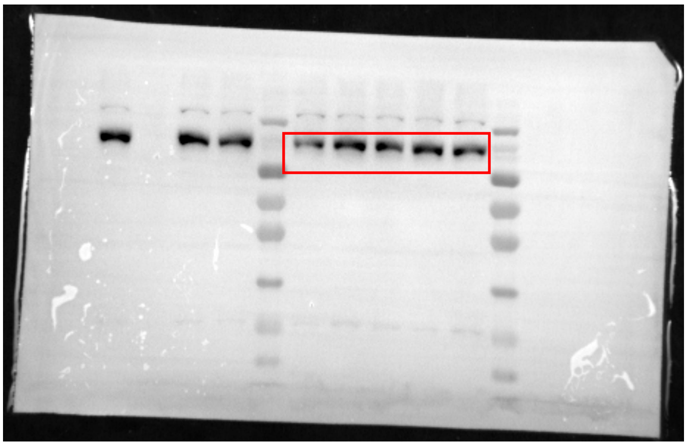

4. Input: Myc-YOD1

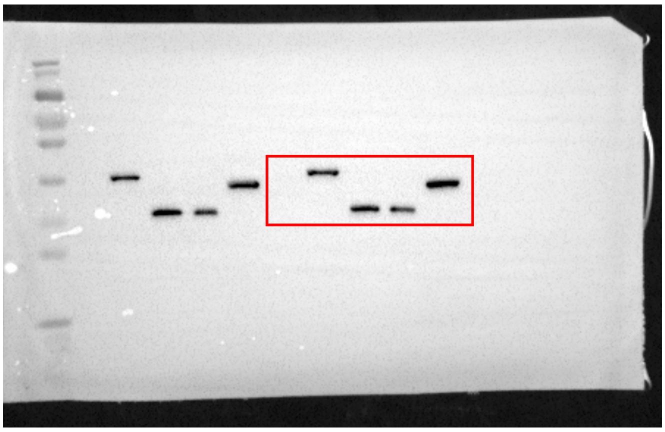

5. Actin

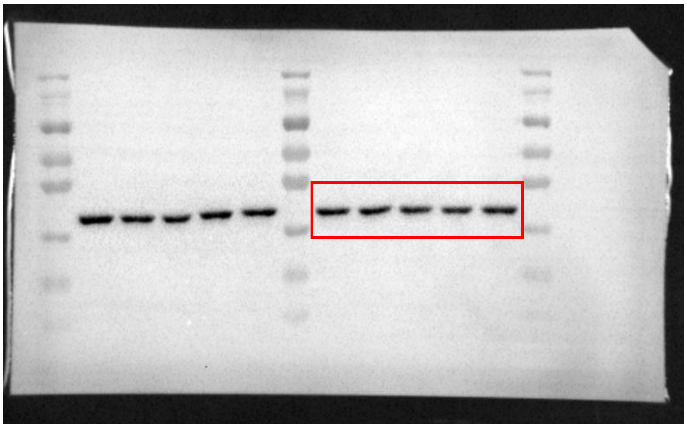

Figure 3 H

1. IP: Flag-NLRP3

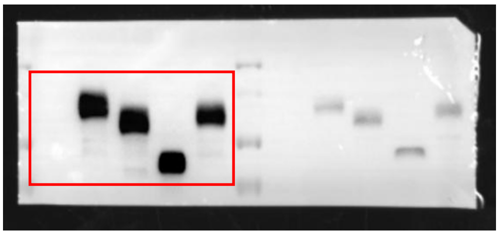

2. IP: Myc-YOD1

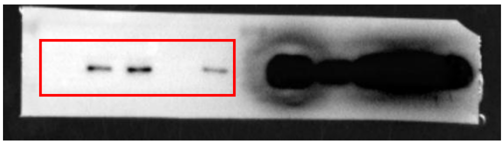

3. Input: Flag-NLRP3

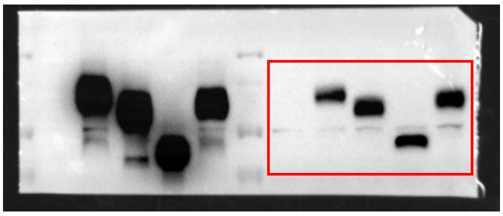

4. Input: Myc-YOD1

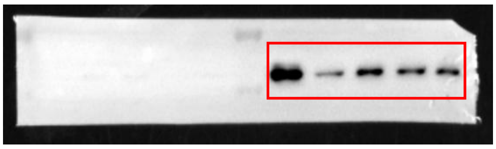

5. Actin

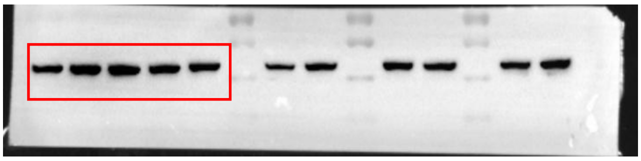

Figure 4 A

1. p20

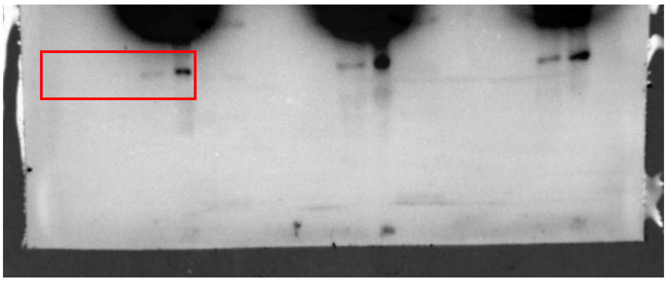

2. p17

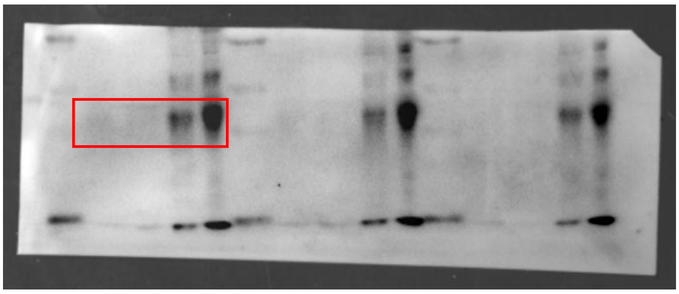

3. NLRP3

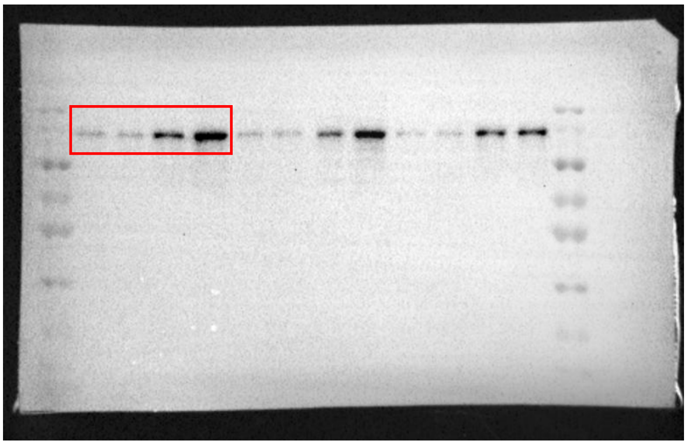

4. Caspase-1

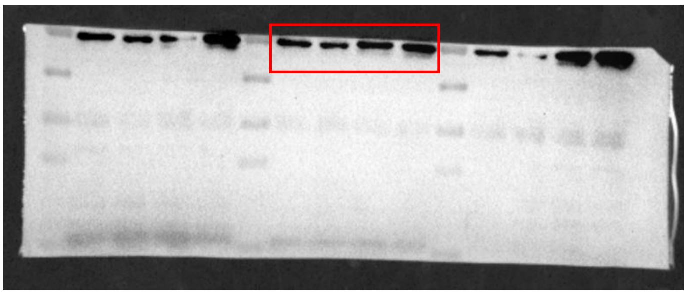

5. IL-1 β

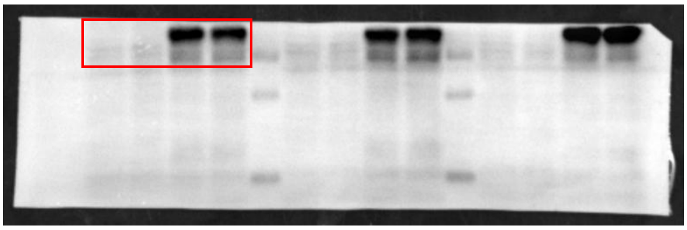

6. YOD1

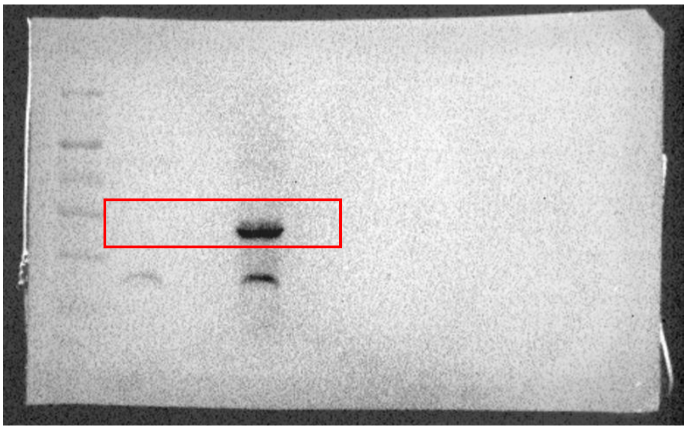

7. Actin

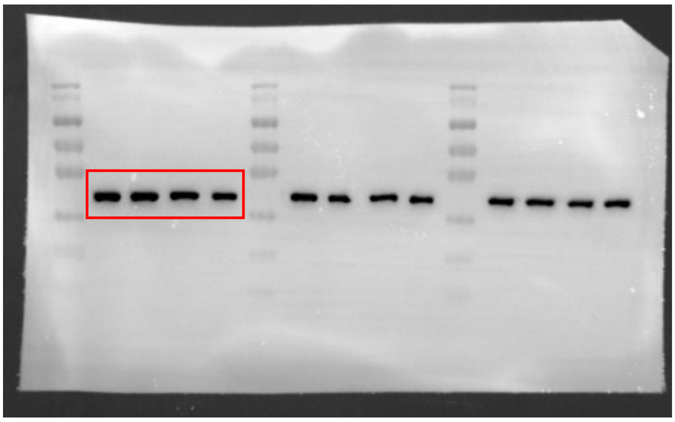

Figure 4 F

1. NLRP3

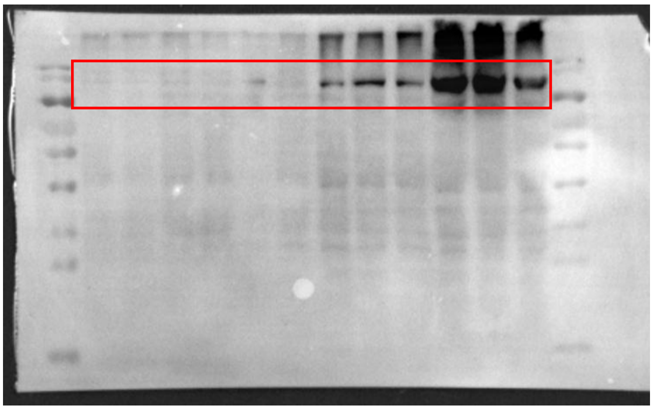

2. Actin

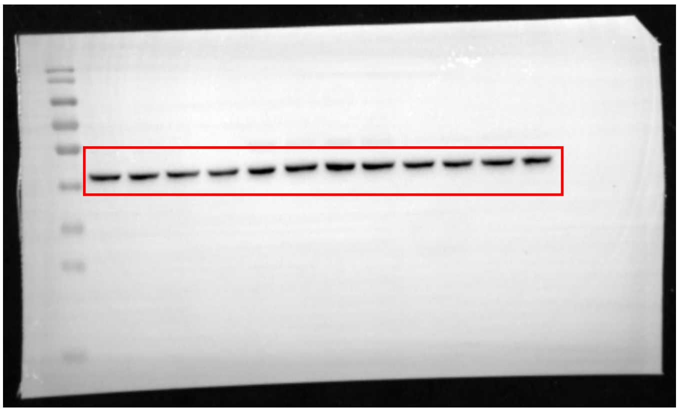

Figure 6 A

1. IP: HA-Ub-WT

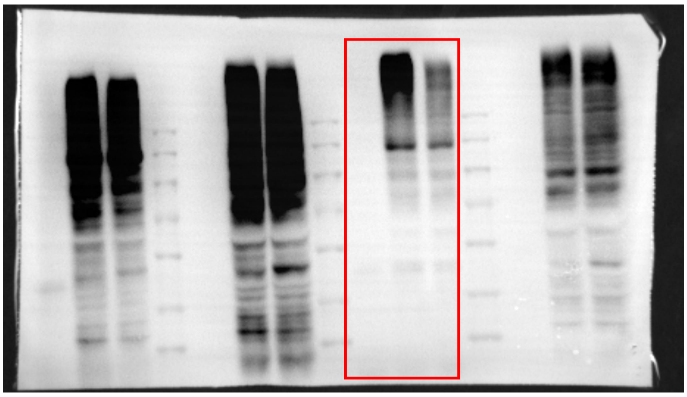

2. IP: Flag-NLRP3

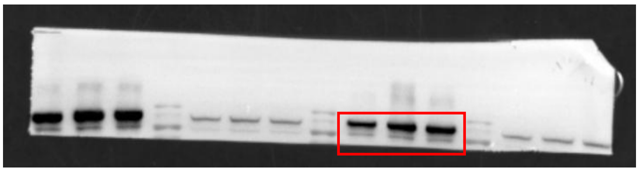

3. Input: HA-Ub-WT

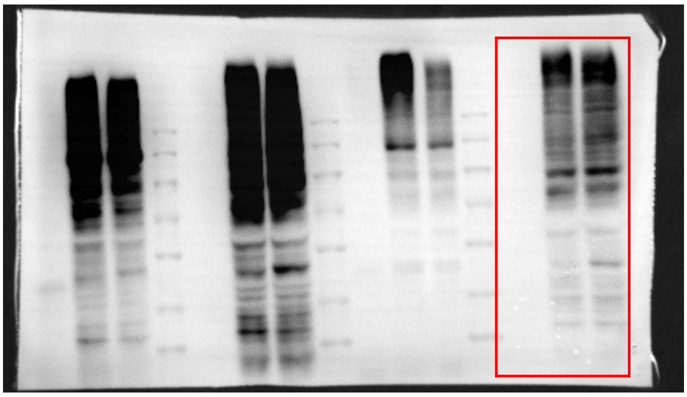

4. Input: Flag-NLRP3

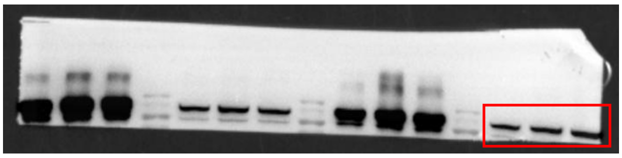

5. Input: Myc-YOD1

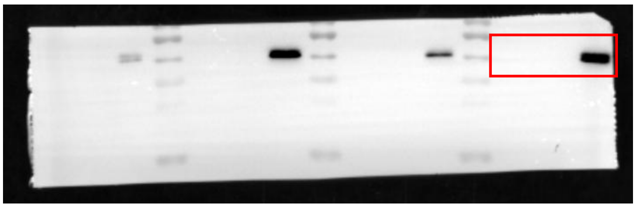

6. Actin

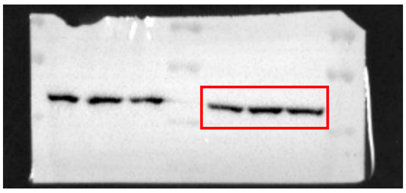

Figure 6 B

1. IP: Ub

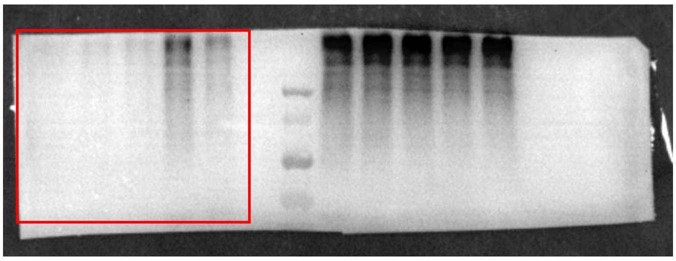

2. IP: NLRP3

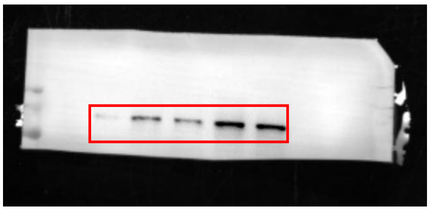

3. Input: Ub

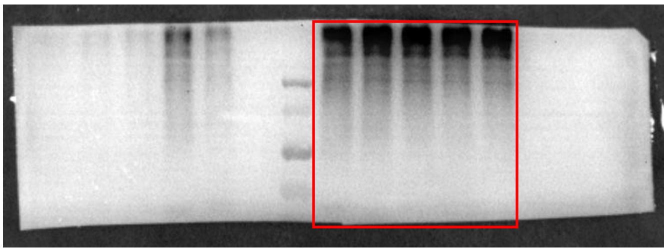

4. Input: NLRP3

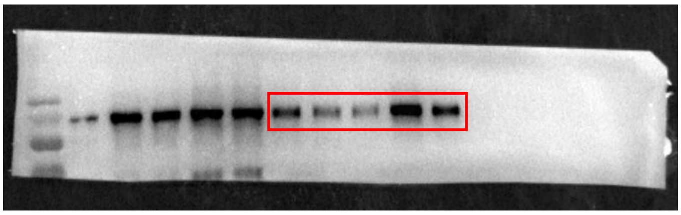

5. Input: Myc-YOD1

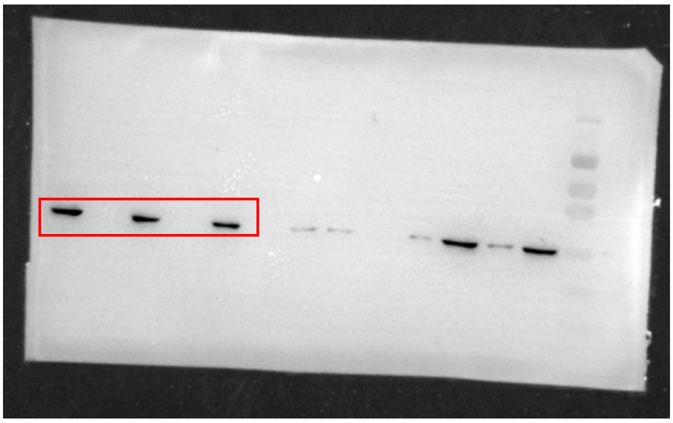

6. Actin

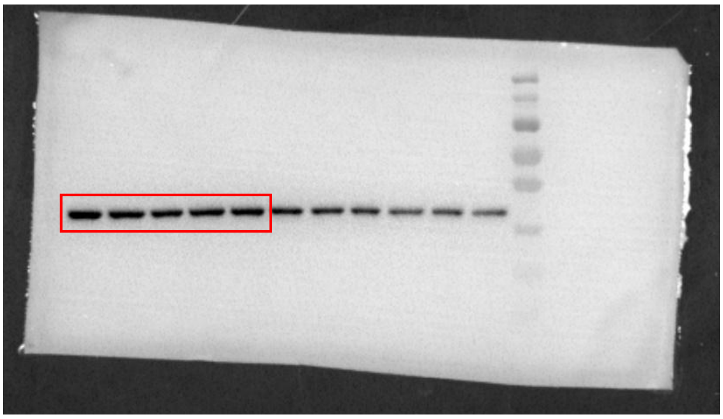

Figure 6 C

1. IP: Ub

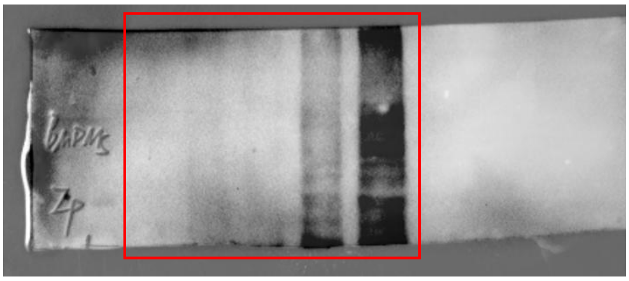

2. IP: NLRP3

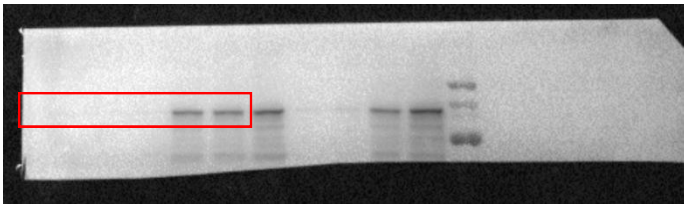

3. Input: Ub

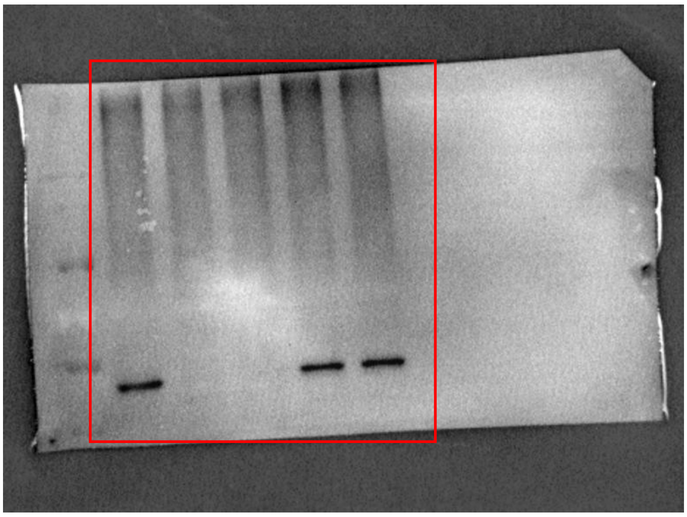

4. Input: NLRP3

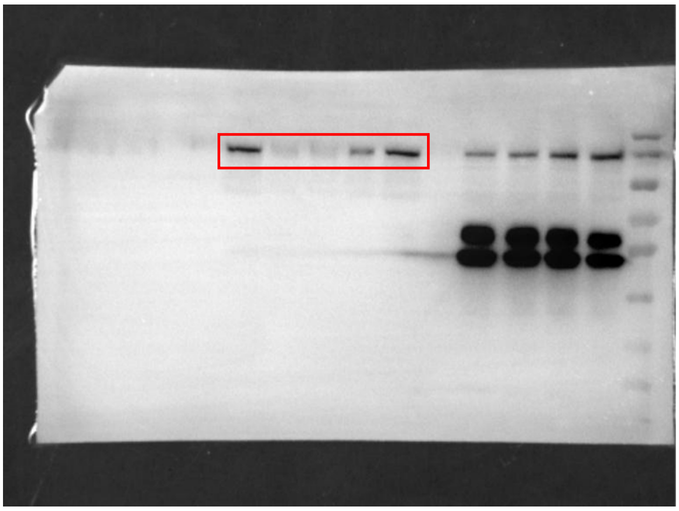

5. Input: YOD1

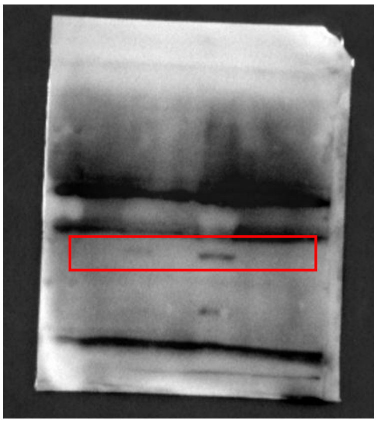

6. Actin

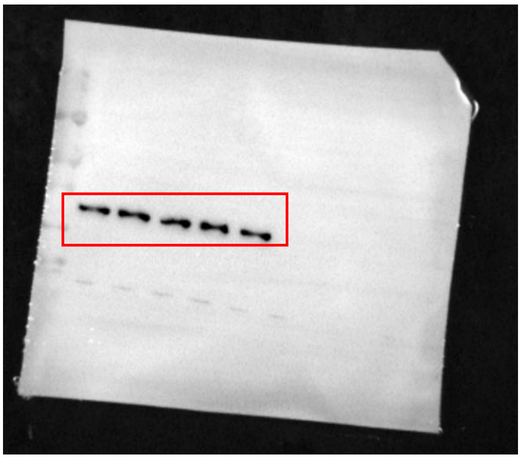

Figure 6 D

1. IP: HA-Ub-K6

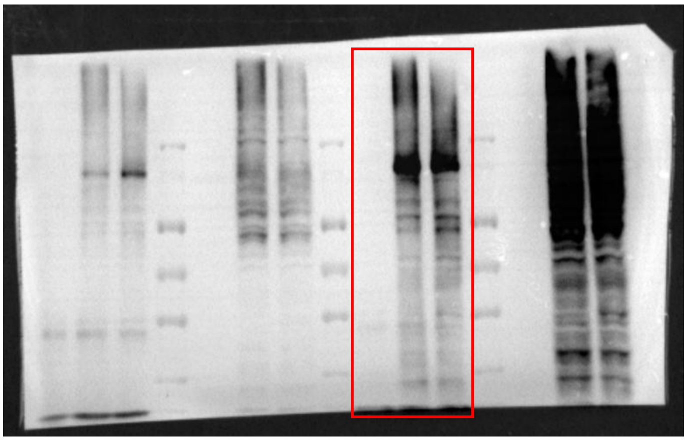

2. IP: Flag-NLRP3

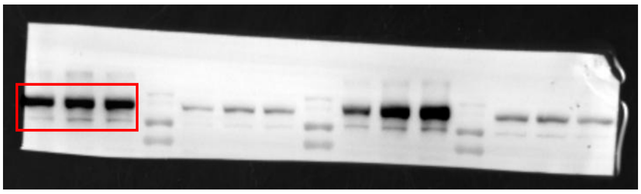

3. Input: HA-Ub-K6

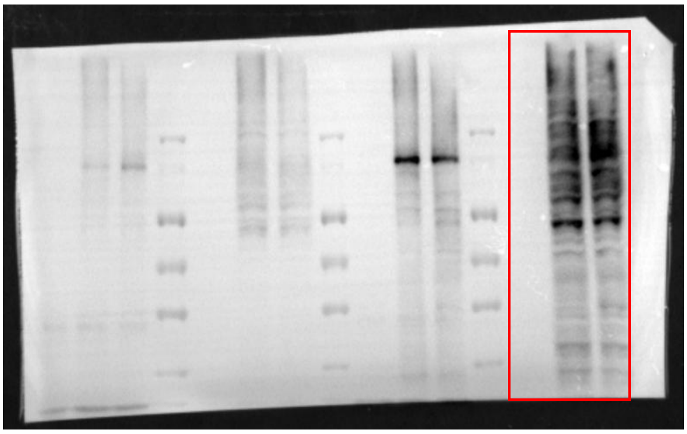

4. Input: Flag-NLRP3

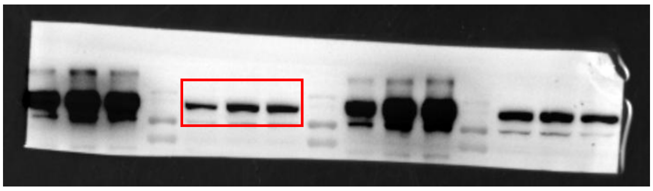

5. Input: Myc-YOD1

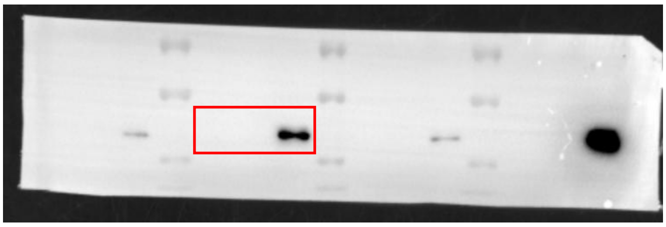

6. Actin

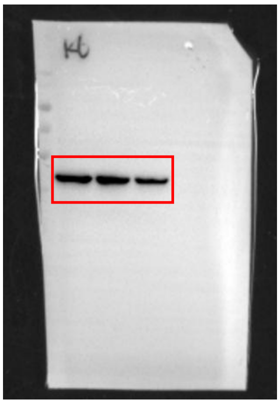

Figure 6 E

1. IP: HA-Ub-K11

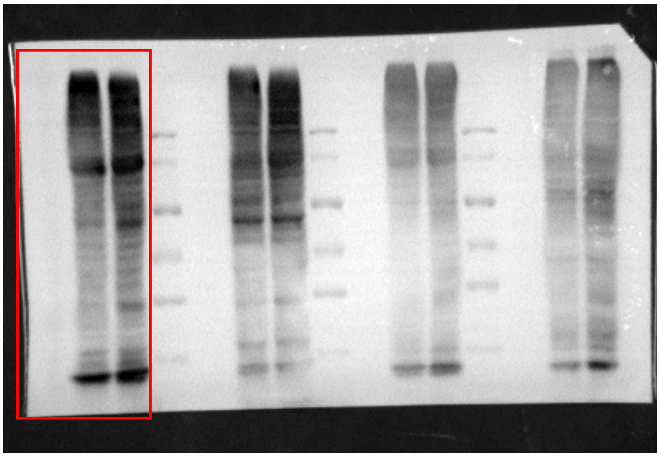

2. IP: Flag-NLRP3

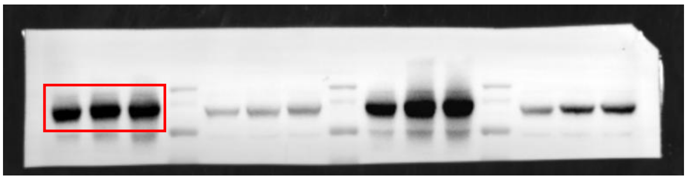

3. Input: HA-Ub-K11

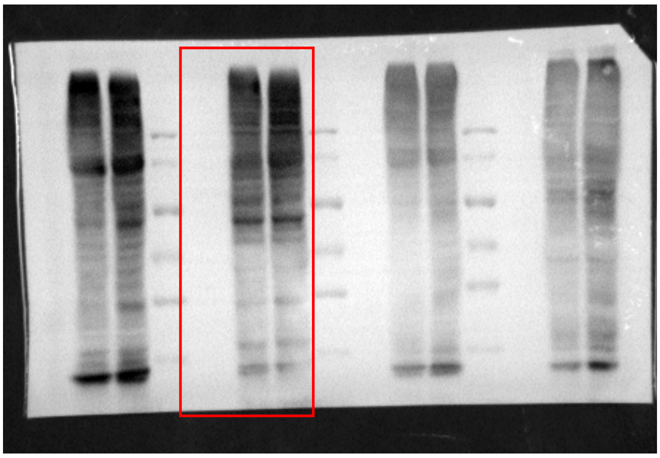

4. Input: Flag-NLRP3

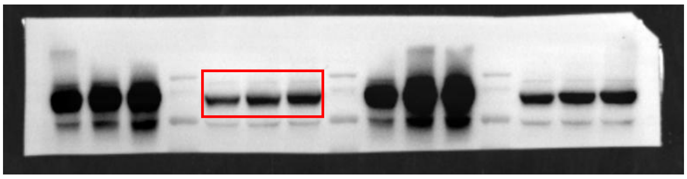

5. Input: Myc-YOD1

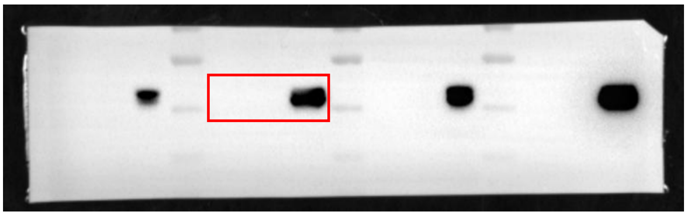

6. Actin

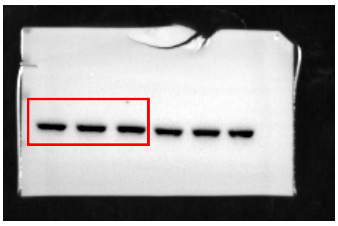

Figure 6 F

1. IP: HA-Ub-K27

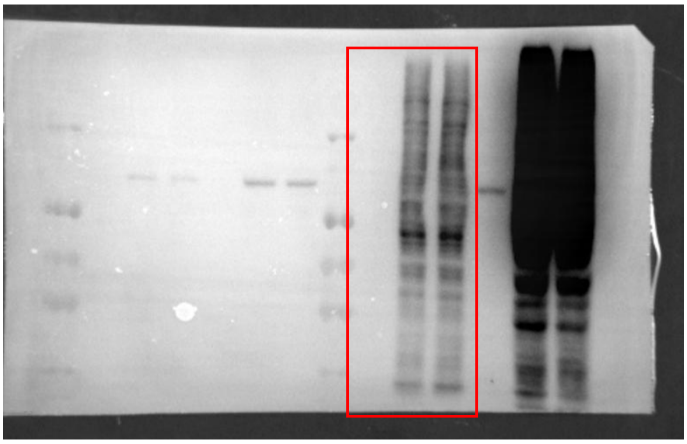

2. IP: Flag-NLRP3

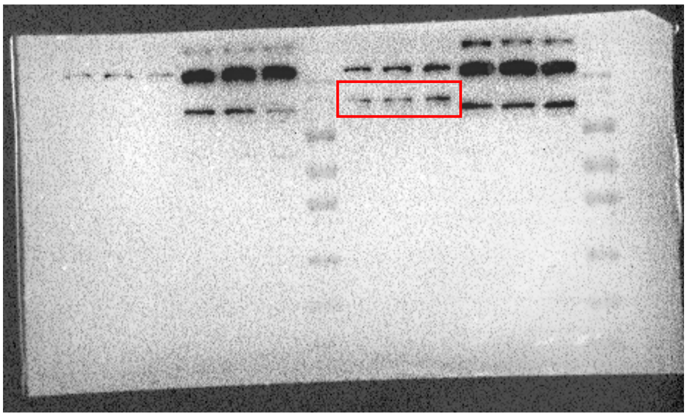

3. Input: HA-Ub-K27

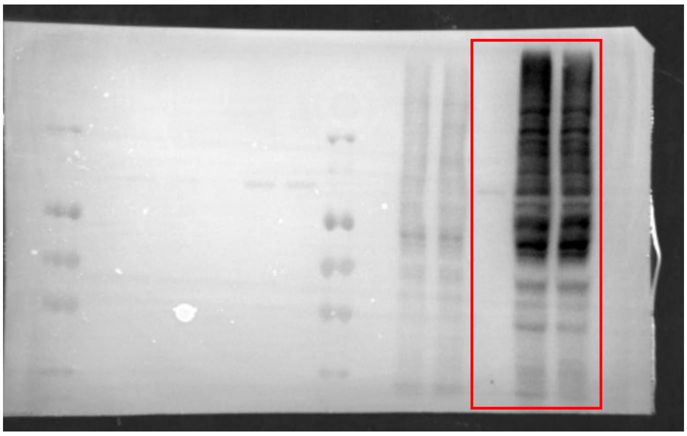

4. Input: Flag-NLRP3

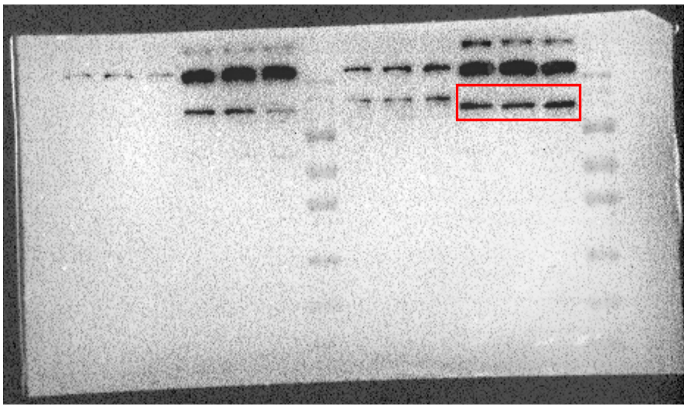

5. Input: Myc-YOD1

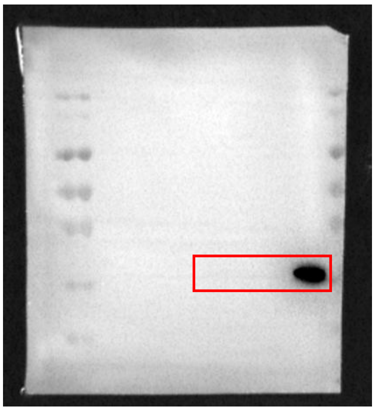

6. Actin

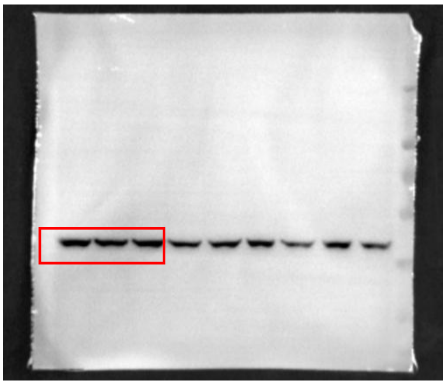

Figure 6 G

1. IP: HA-Ub-K29

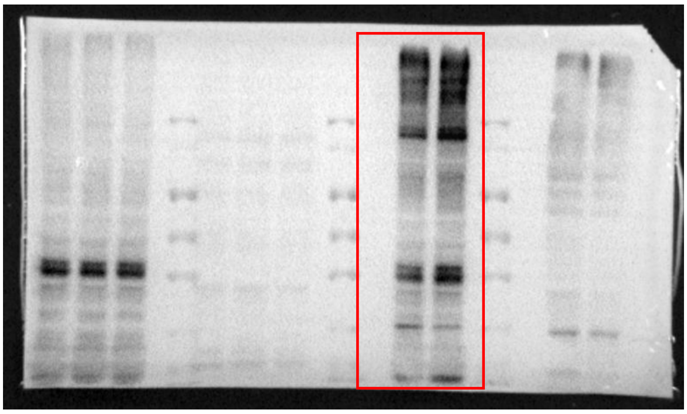

2. IP: Flag-NLRP3

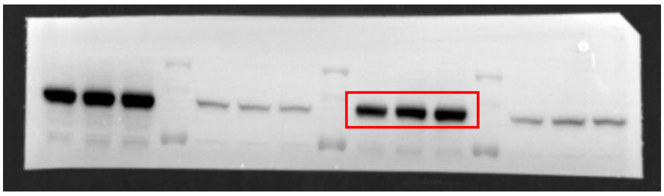

3. Input: HA-Ub-K29

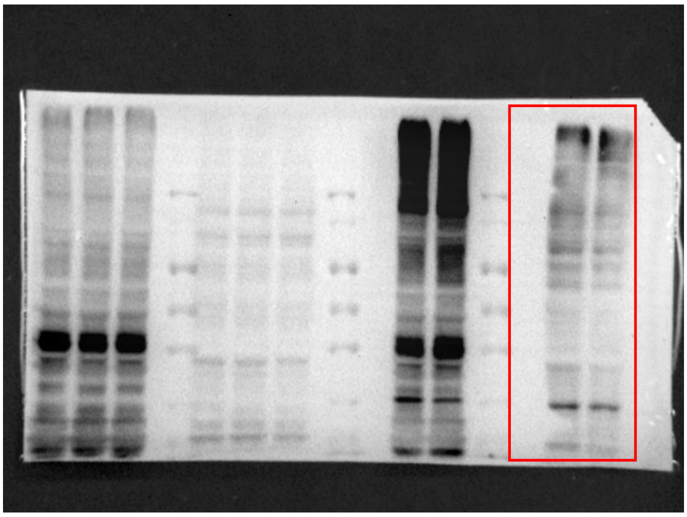

4. Input: Flag-NLRP3

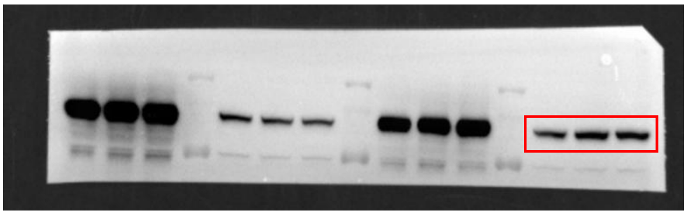

5. Input: Myc-YOD1

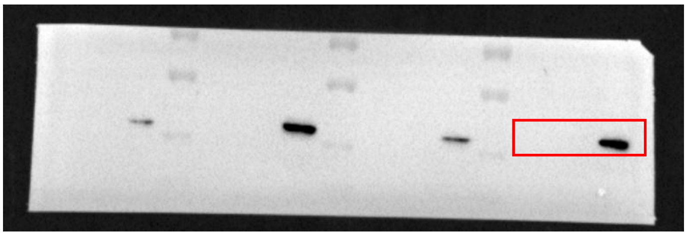

6. Actin

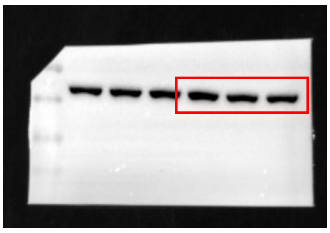

Figure 6 H

1. IP: HA-Ub-K33

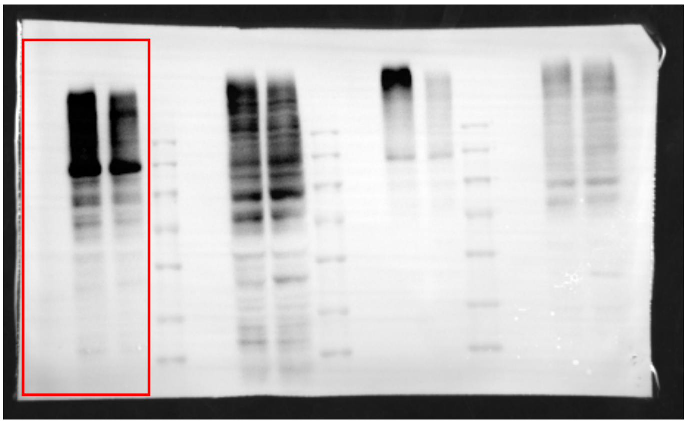

2. IP: Flag-NLRP3

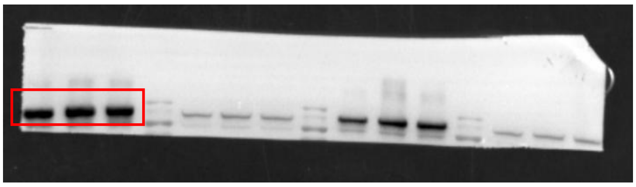

3. Input: HA-Ub-K33

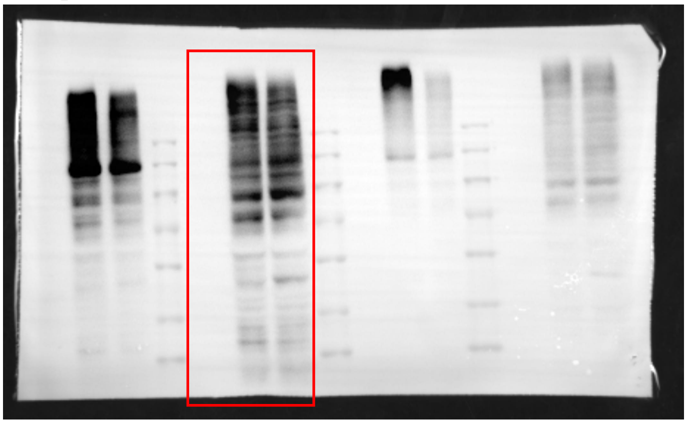

4. Input: Flag-NLRP3

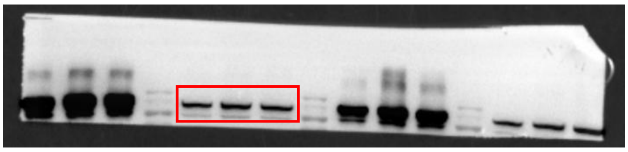

5. Input: Myc-YOD1

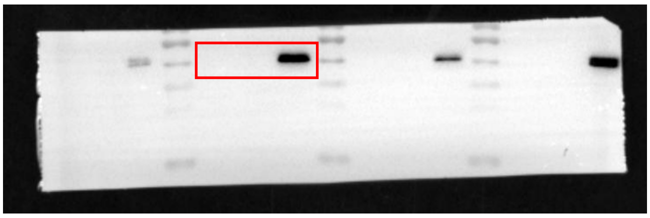

6. Actin

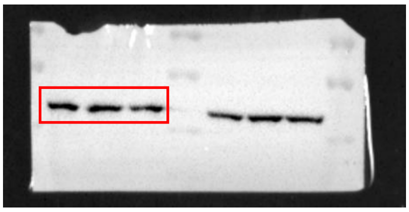

Figure 6 I

1. IP: HA-Ub-K48

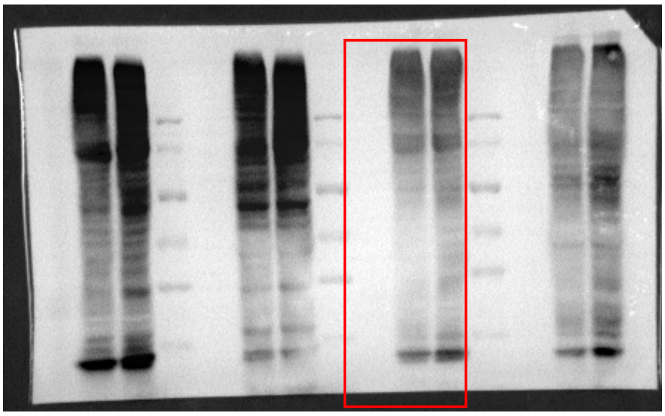

2. IP: Flag-NLRP3

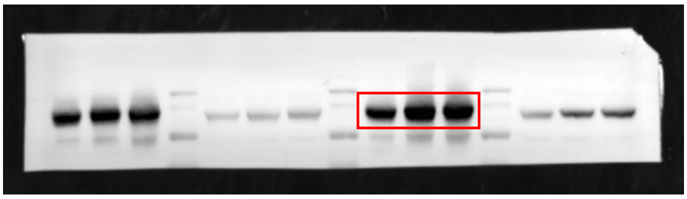

3. Input: HA-Ub-K48

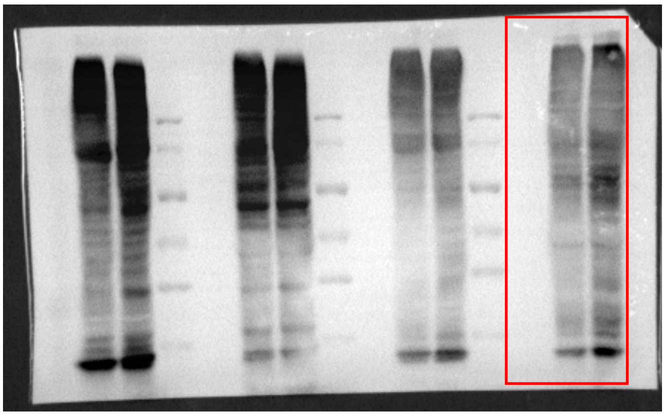

4. Input: Flag-NLRP3

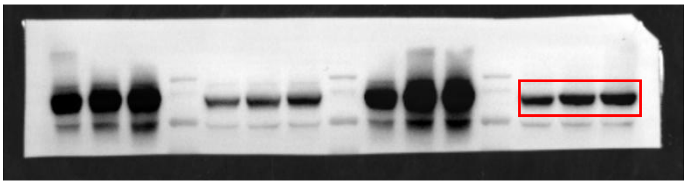

5. Input: Myc-YOD1

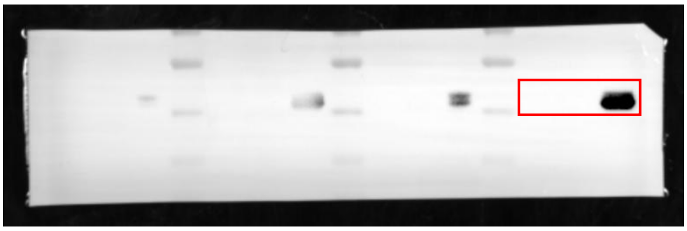

6. Actin

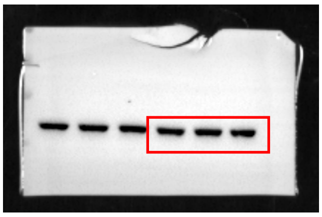

Figure 6 J

1. IP: HA-Ub-K63

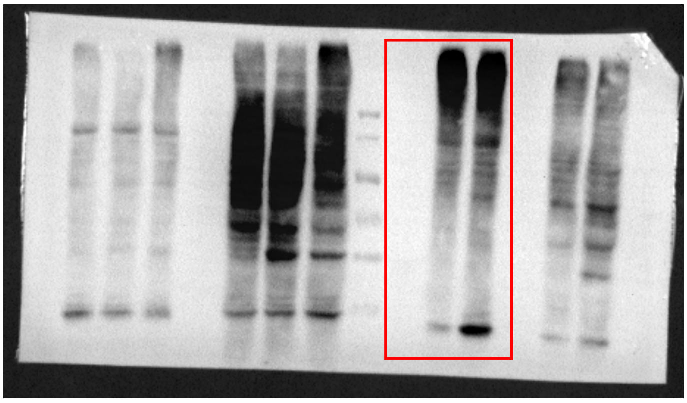

2. IP: Flag-NLRP3

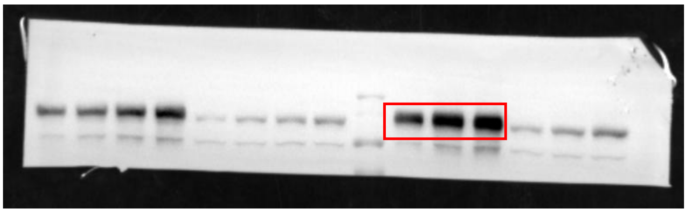

3. Input: HA-Ub-K63

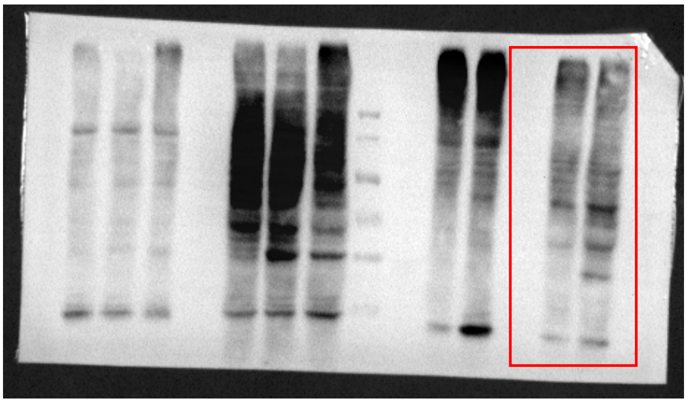

4. Input: Flag-NLRP3

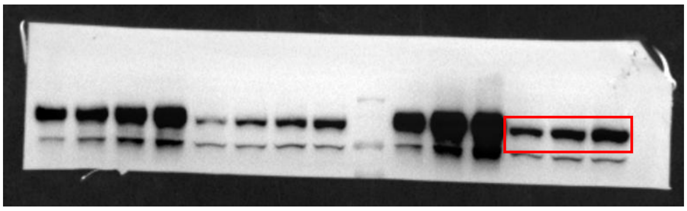

5. Input: Myc-YOD1

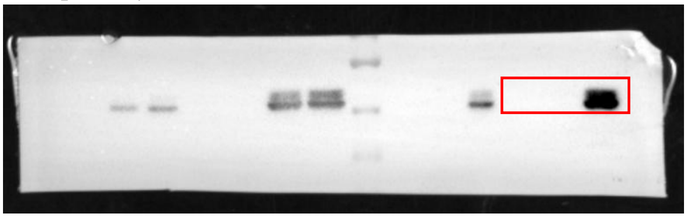

6. Actin

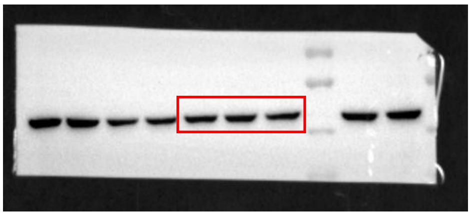

Figure 7 B

1. IP: Myc-YOD1

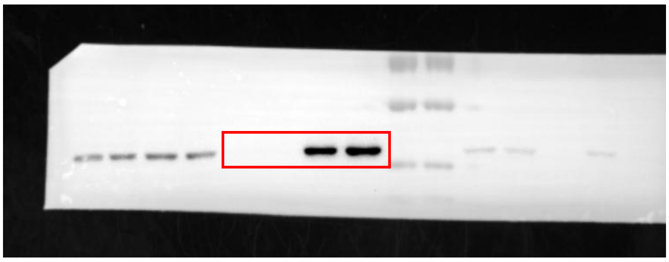

2. IP: Flag-NLRP3

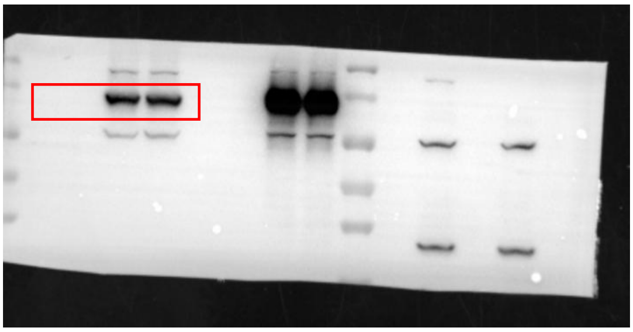

3. Input: Myc-YOD1

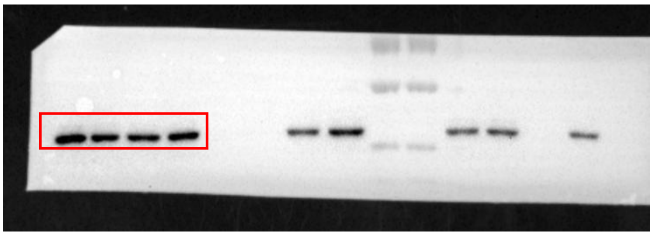

4. Input: Flag-NLRP3

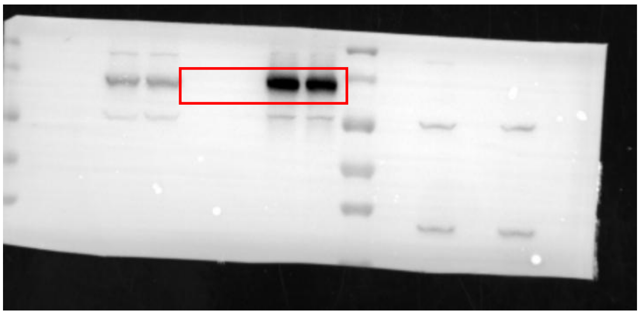

5. ACTIN

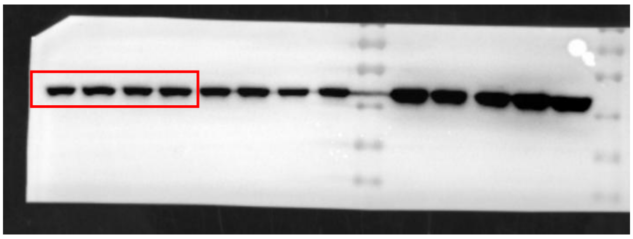

Figure 7 C

1. IP: Flag-NLRP3

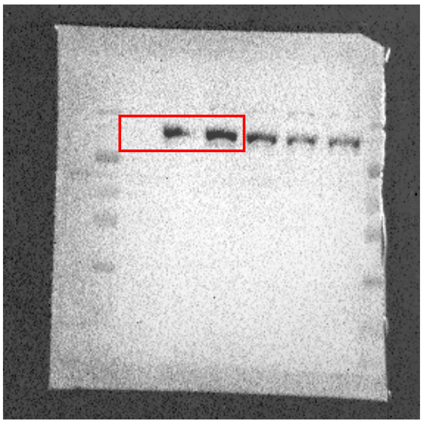

2. IP: Myc-YOD1

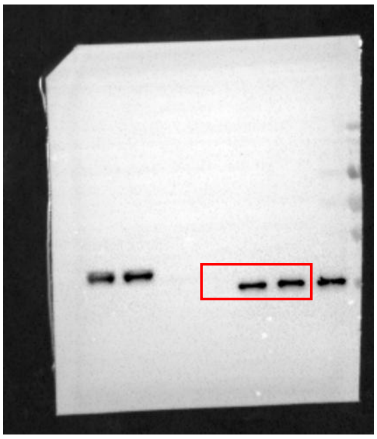

3. Input: Flag-NLRP3

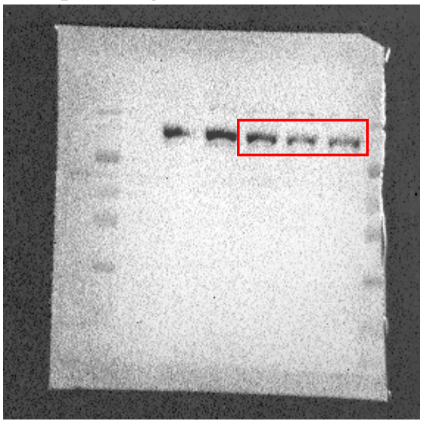

4. Input: Myc-YOD1

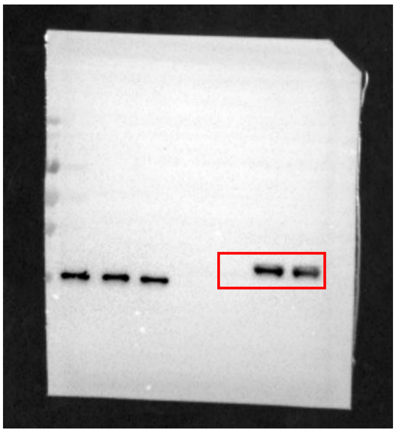

5. ACTIN

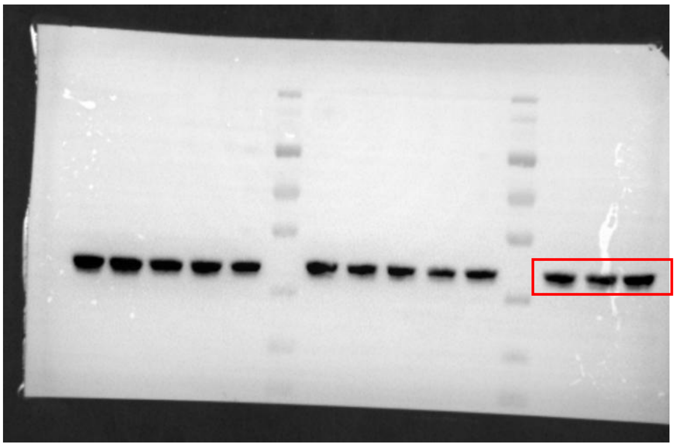

Figure 7 D

1. NLRP3

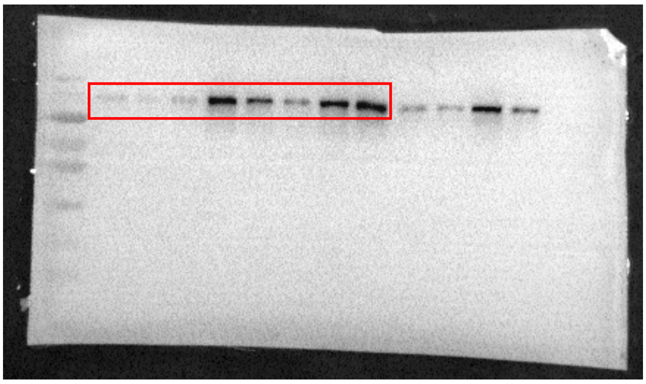

2. Myc-Yod1/Yod1^{C155S}

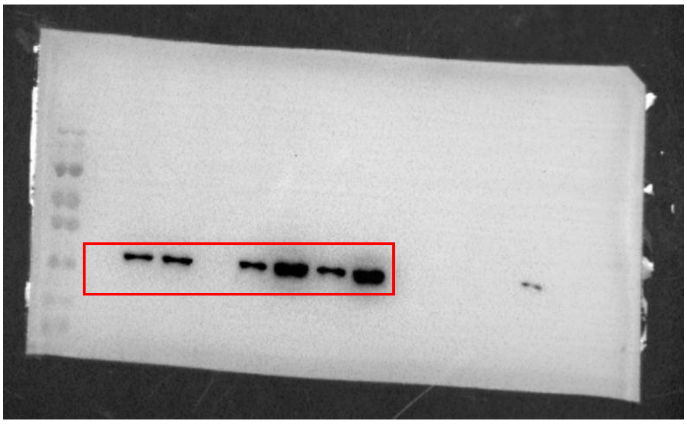

3. ACTIN

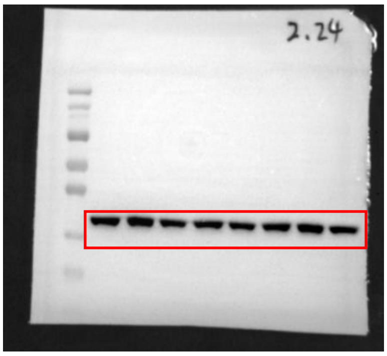

Figure 7 F

1. IP: HA-Ub-WT

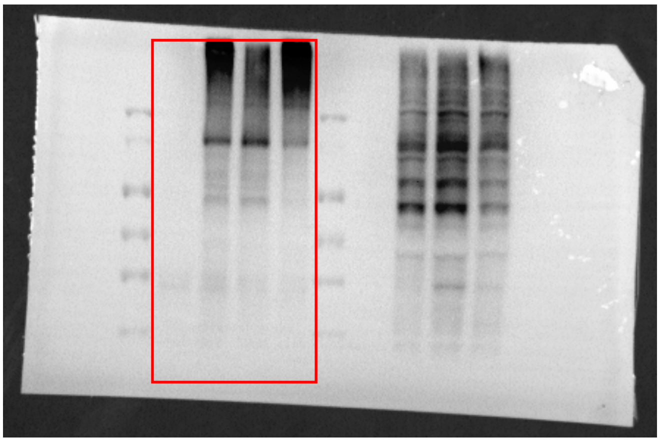

2. IP: Flag-NLRP3

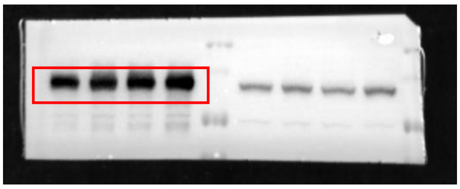

3. Input: HA-Ub-WT

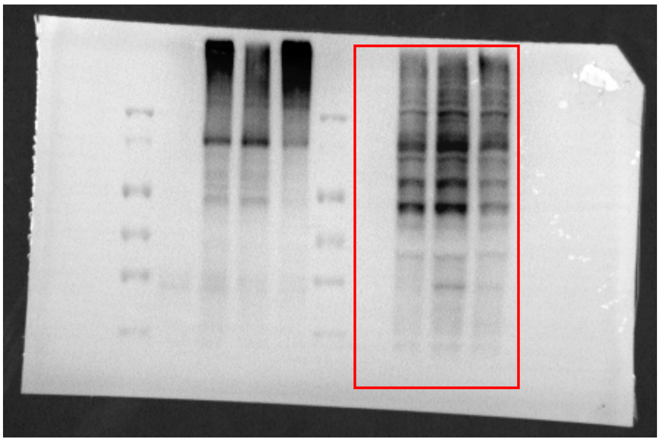

4. Input: Flag-NLRP3

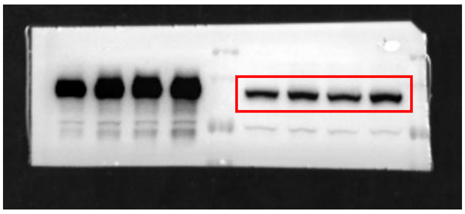

5. Input: Myc-YOD1

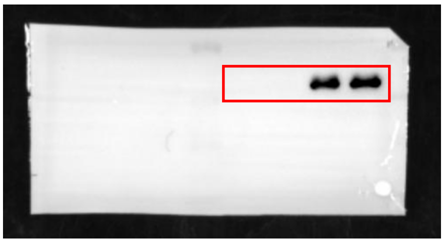

6. ACTIN

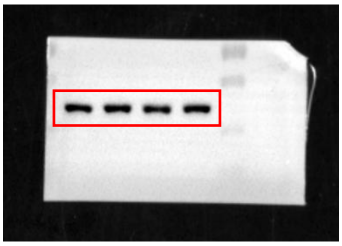

Figure 7 G

1. IP: HA-Ub-K33

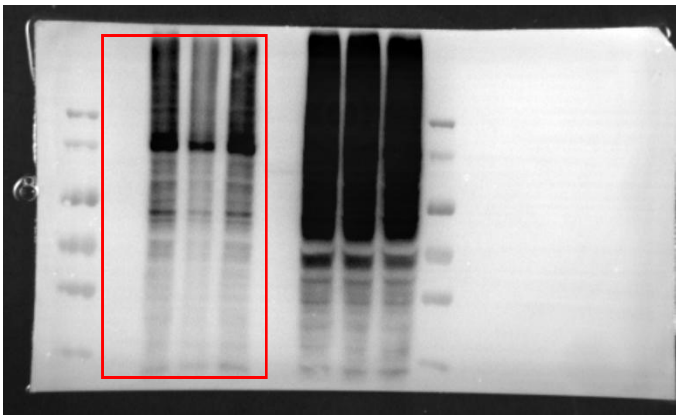

2. IP: Flag-NLRP3

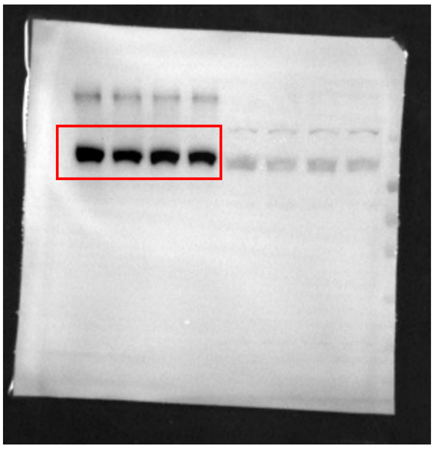

3. Input: HA-Ub-K33

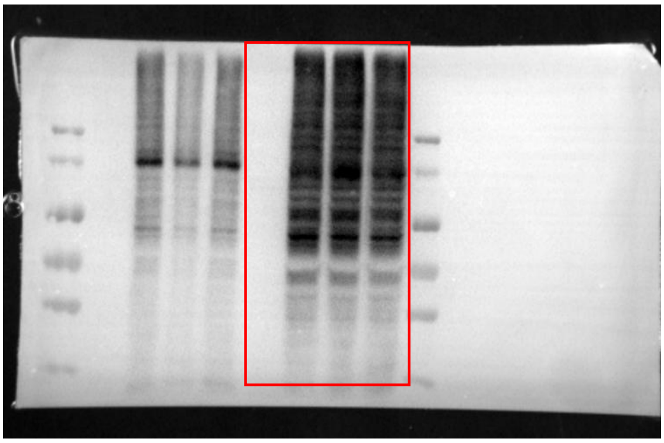

4. Input: Flag-NLRP3

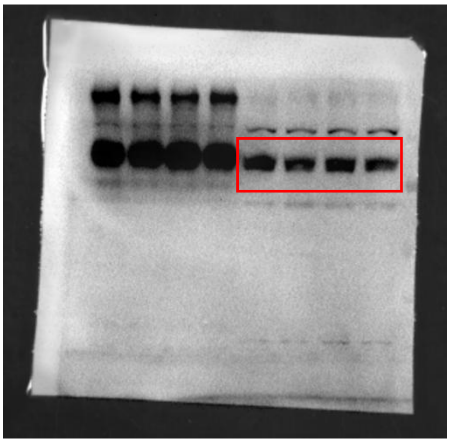

5. Input: Myc-YOD1

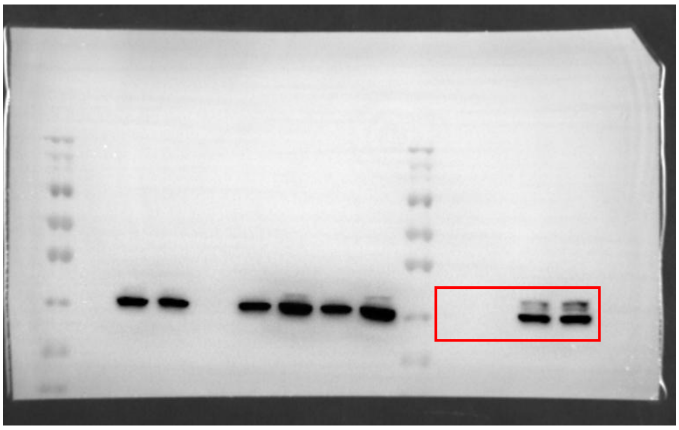

6. ACTIN

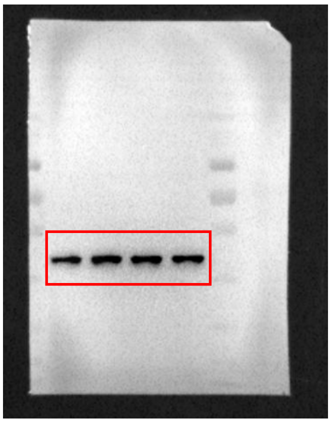

Figure S1 B

1. YOD1

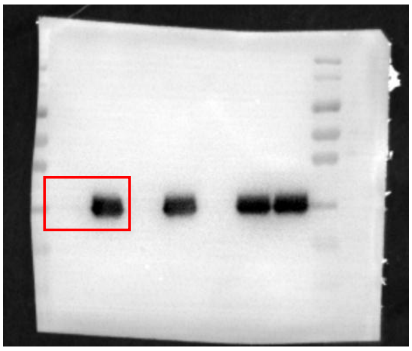

2. ACTIN

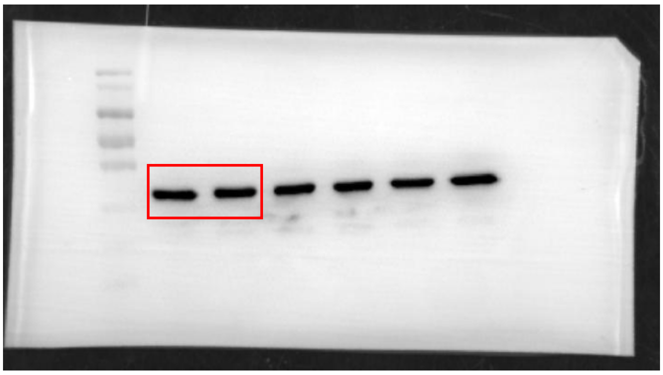

Figure S1 E

1. YOD1

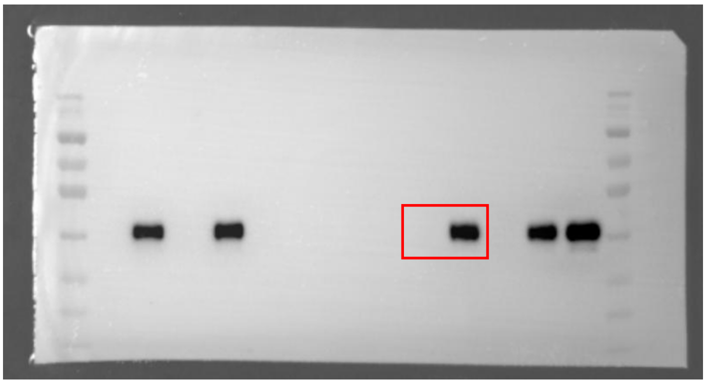

2. ACTIN

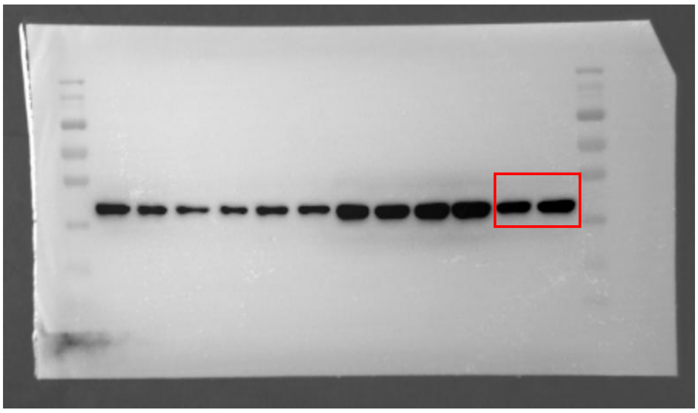

Figure S1 H

1. YOD1

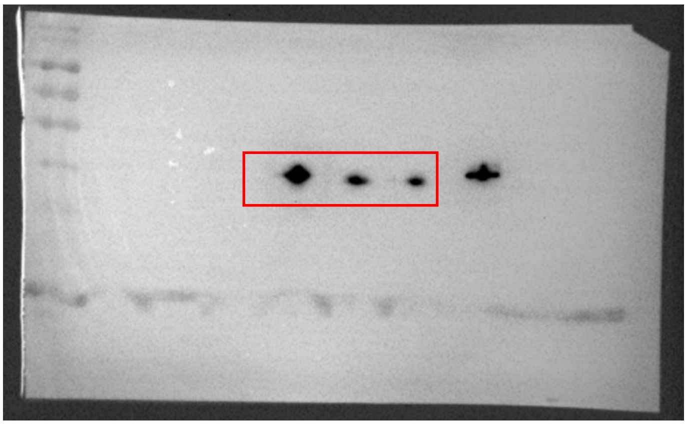

2. ACTIN

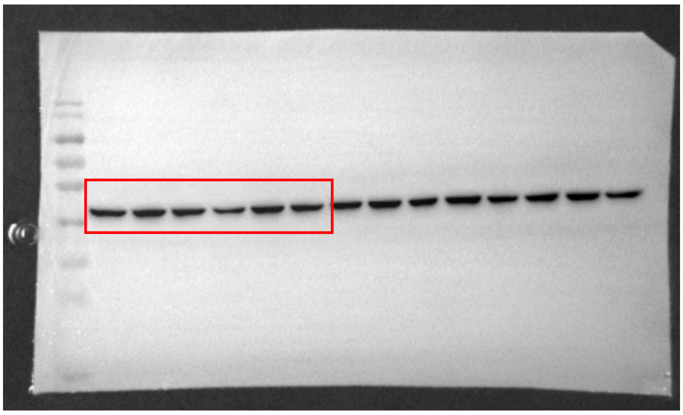

Figure S2 B

1. IP: Myc-YOD1

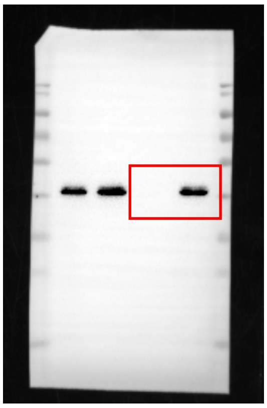

2. IP: Flag-NLRP3

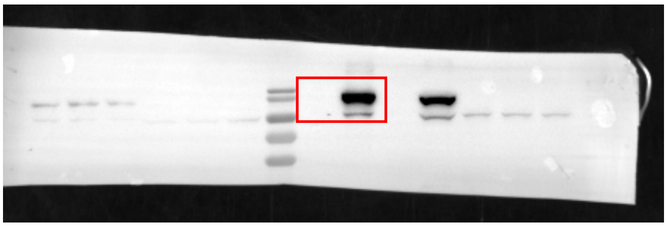

3. Input: Myc-YOD1

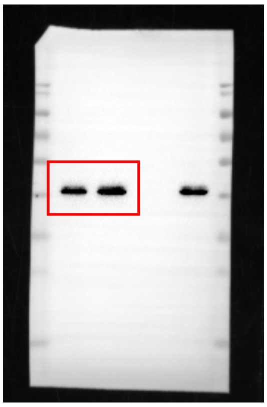

4. Input: Flag-NLRP3

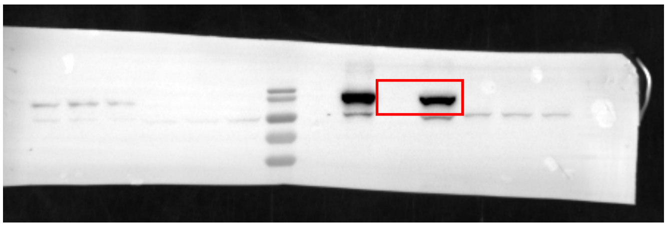

5. ACTIN

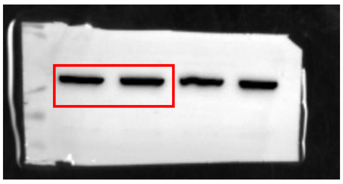

Figure S2 C

1. IP: Myc-YOD1

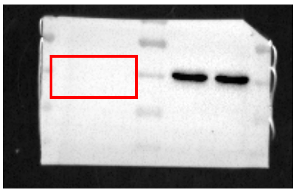

2. IP: Flag-Caspase-1

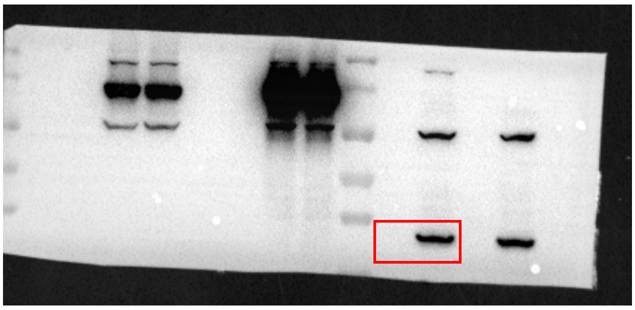

3. Input: Myc-YOD1

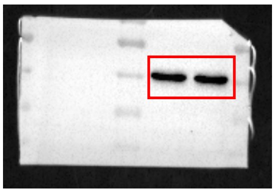

4. Input: Flag-Caspase-1

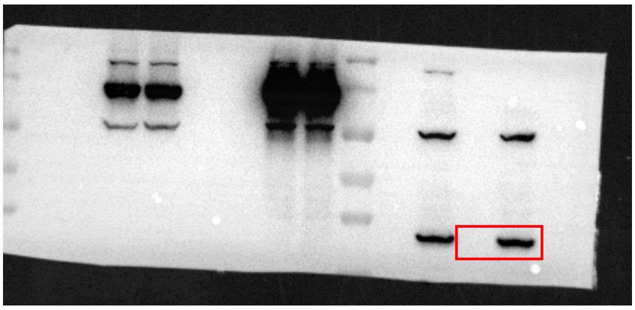

5. ACTIN

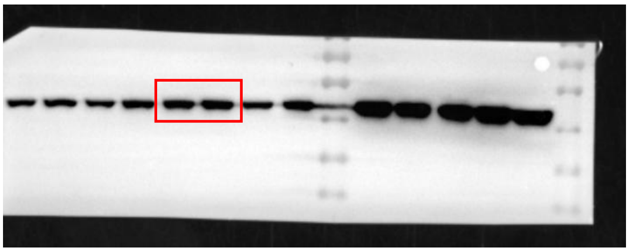

Figure S2 D

1. IP: Myc-YOD1

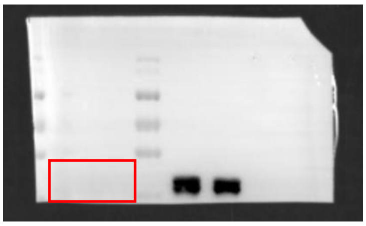

2. IP: Flag-ASC

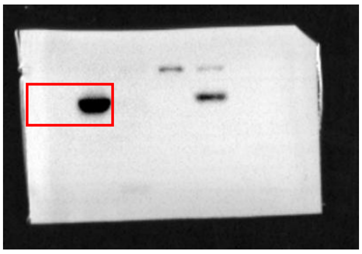

3. Input: Myc-YOD1

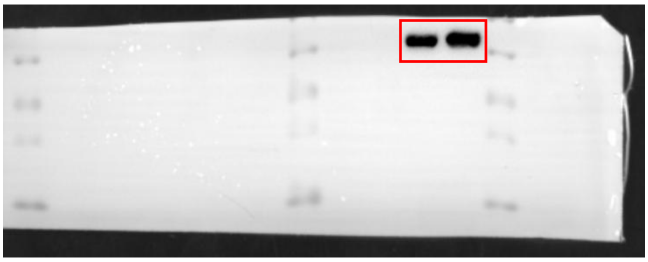

4. Input: Flag-ASC

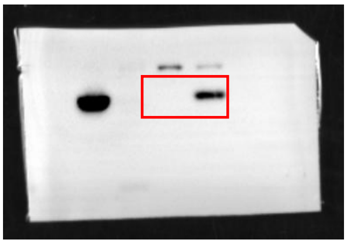

5. ACTIN

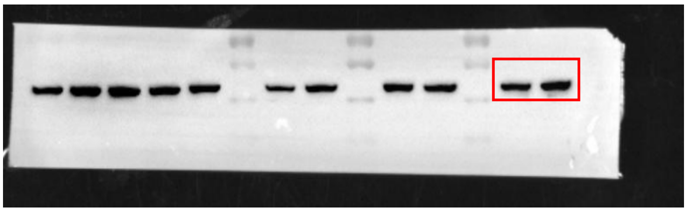

Figure S2 E

1. IP: YOD1

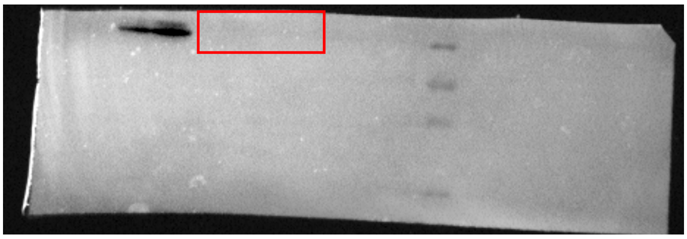

2. IP: Caspase-1

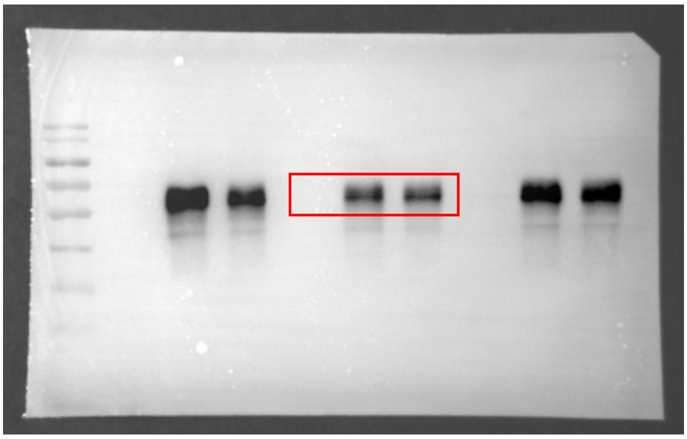

3. Input: YOD1

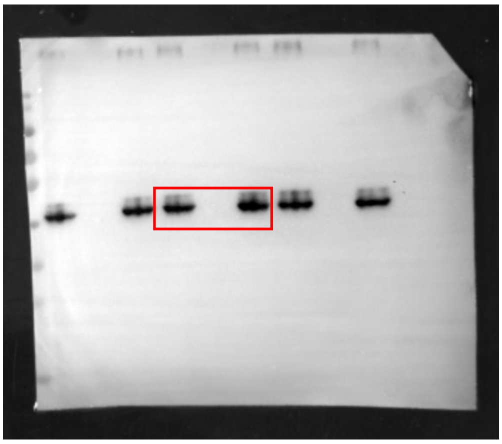

4. Input: Caspase-1

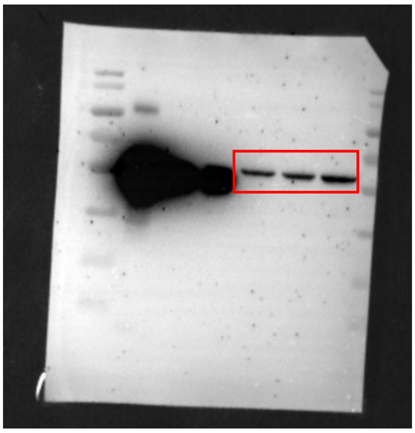

5. ACTIN

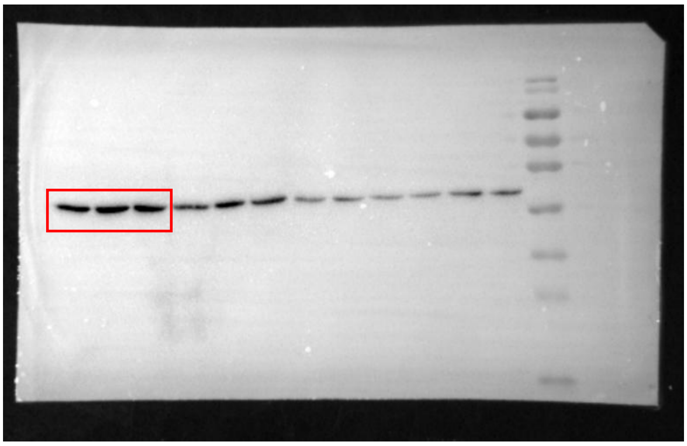

Figure S2 F

1. IP: YOD1

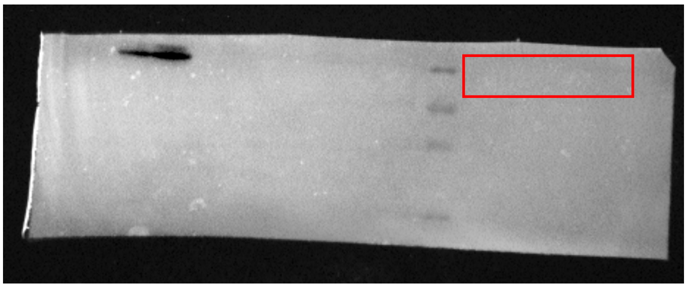

2. IP: ASC

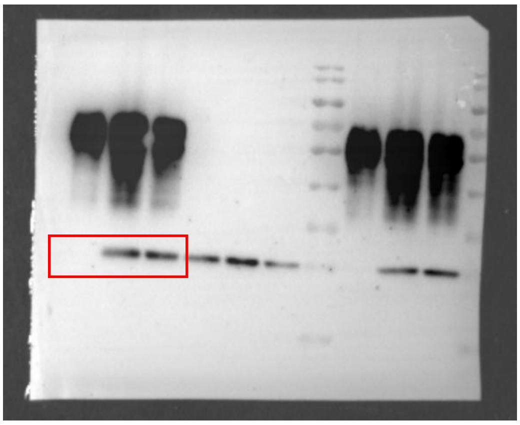

3. Input: YOD1

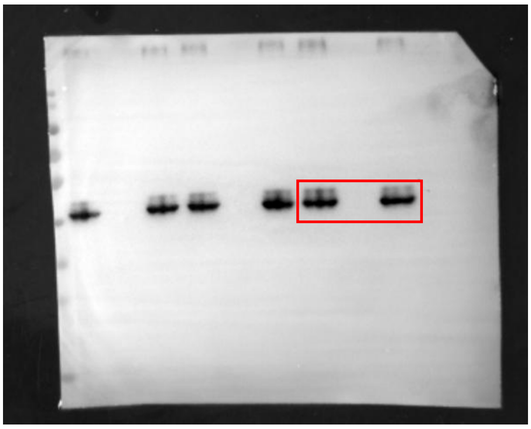

4. Input: ASC

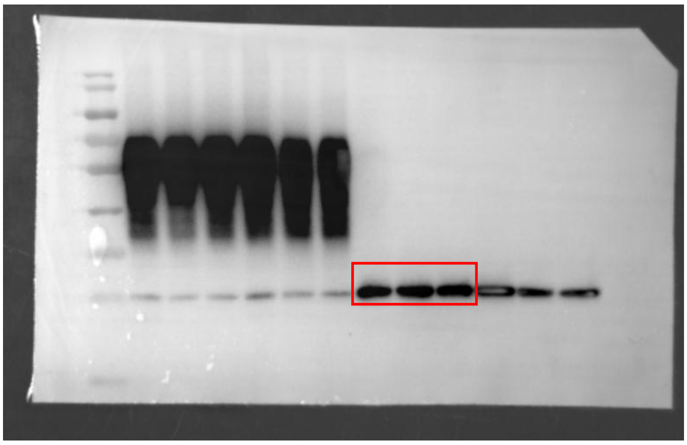

5. ACTIN

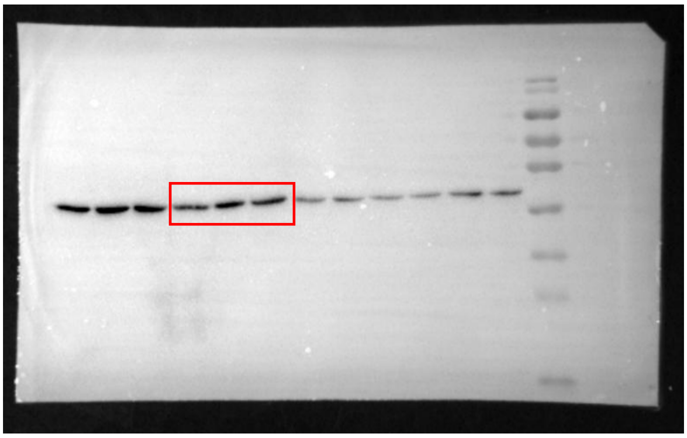

Figure S2 G

1. IP: YOD1

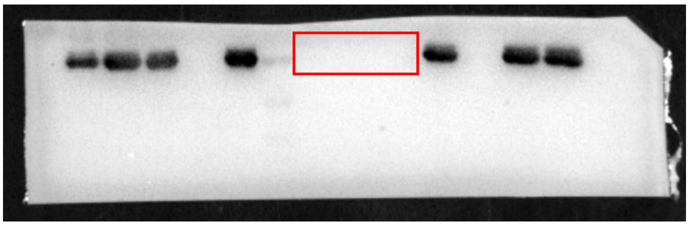

2. IP: Caspase-1

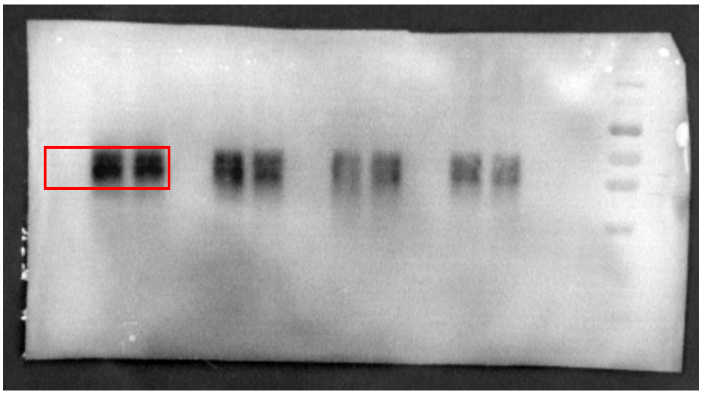

3. Input: YOD1

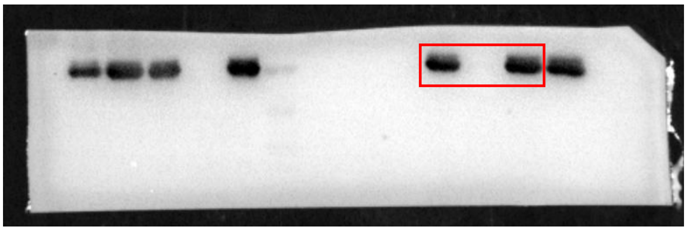

4. Input: Caspase-1

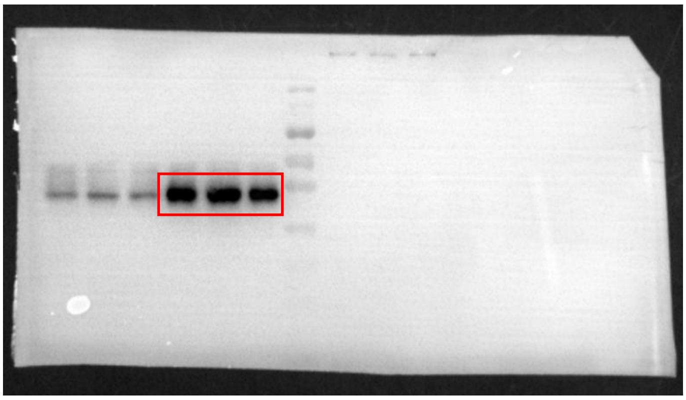

5. ACTIN

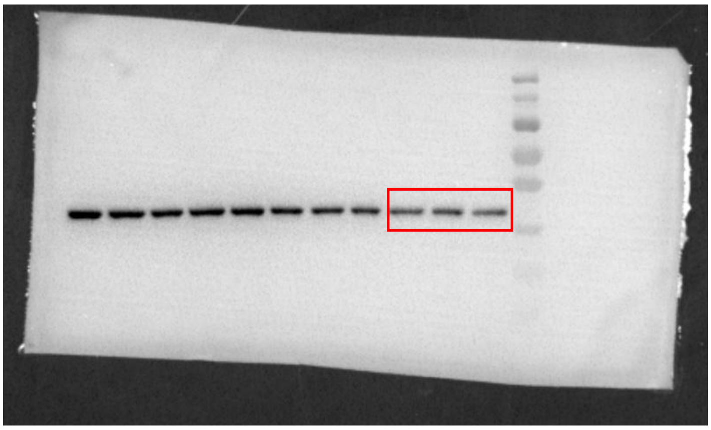

Figure S2 H

1. IP: YOD1

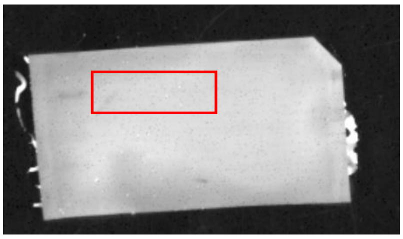

2. IP: ASC

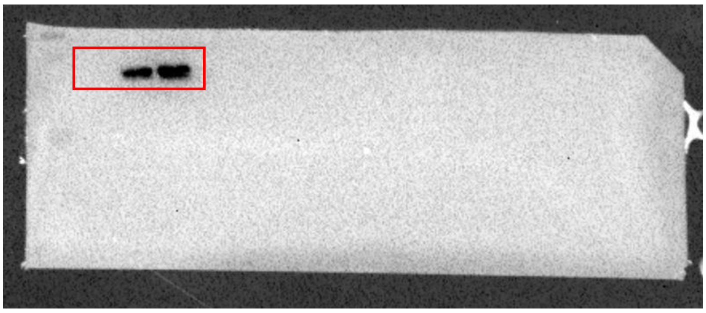

3. Input: YOD1

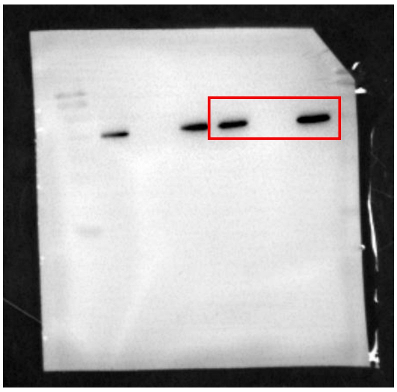

4. Input: ASC

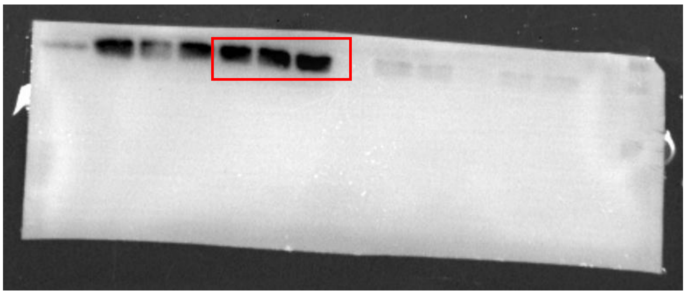

5. ACTIN

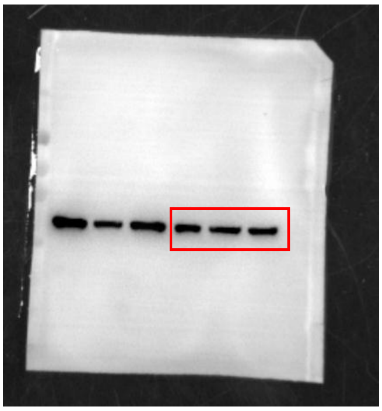

Figure S4 A

1. NLRP3

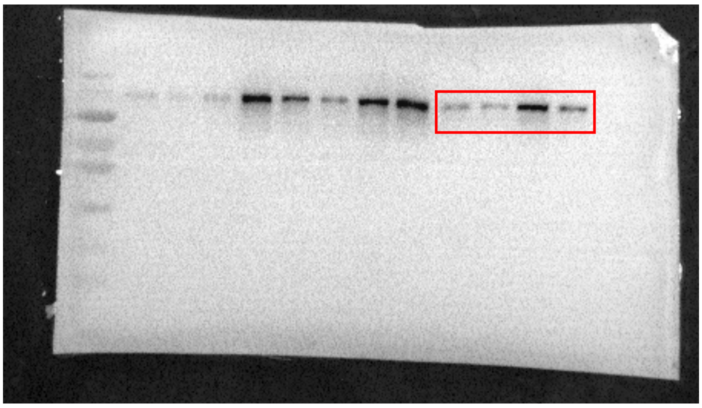

2. Myc-Yod1

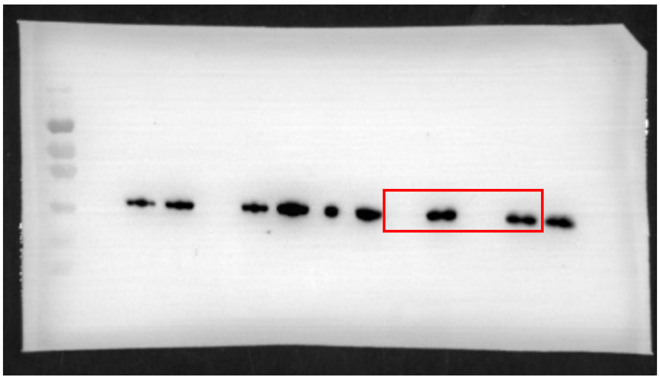

3. ACTIN

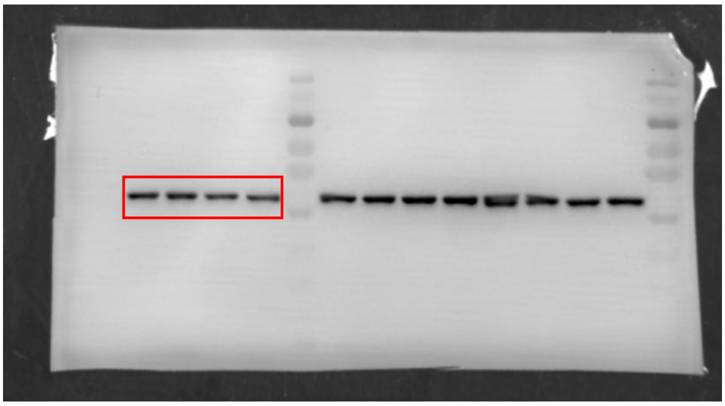

Figure S4 D

1. IP: HA-Ub-WT

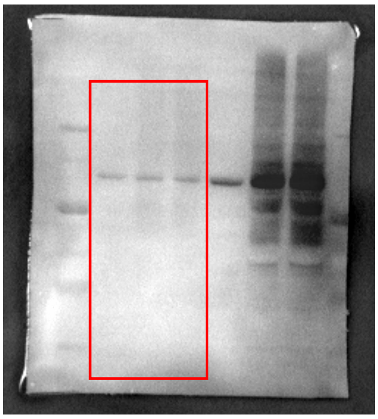

2. IP: Flag-Caspase-1

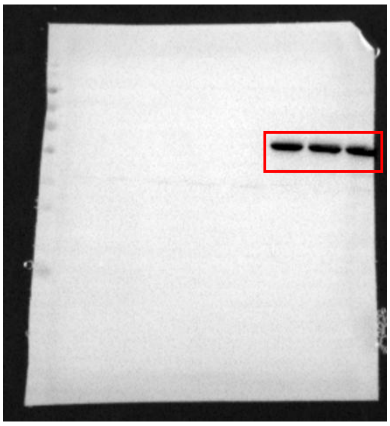

3. Input: HA-Ub-WT

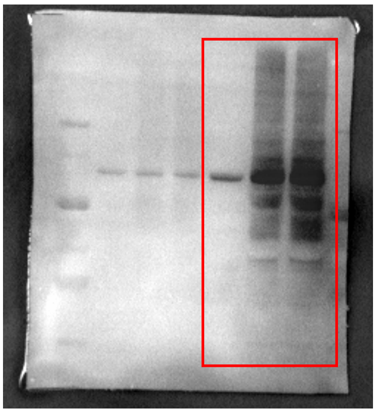

4. Input: Flag-Caspase-1

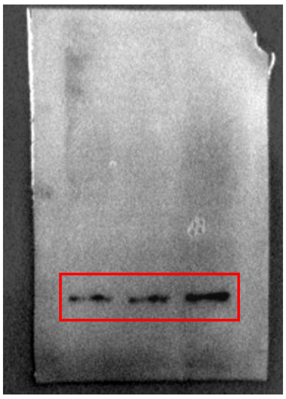

5. Input: Myc-Yod1

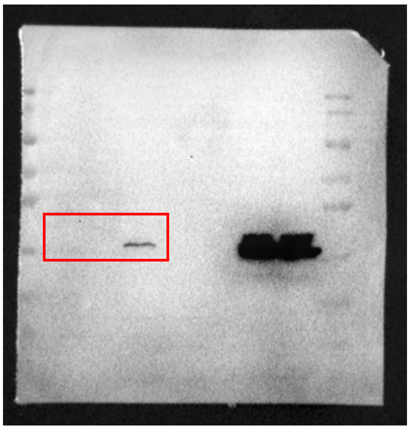

6. ACTIN

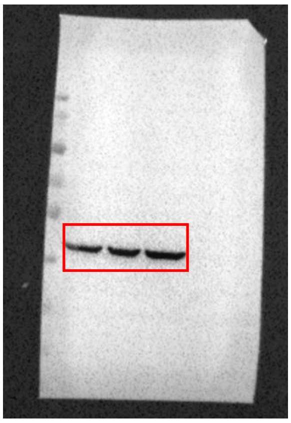

Figure S4 E

1. IP: HA-Ub-WT

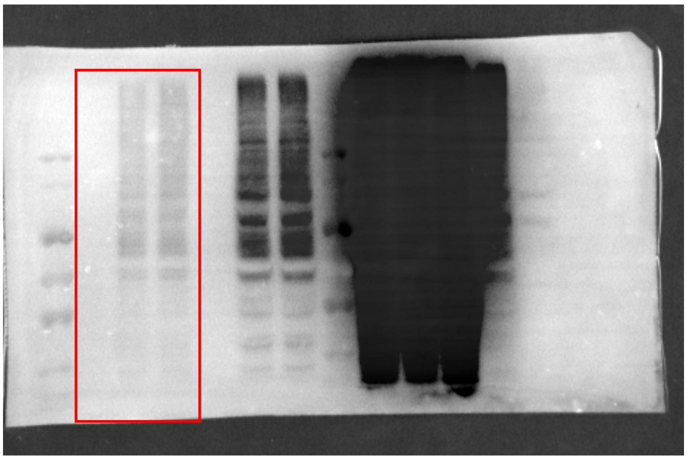

2. IP: Flag-ASC

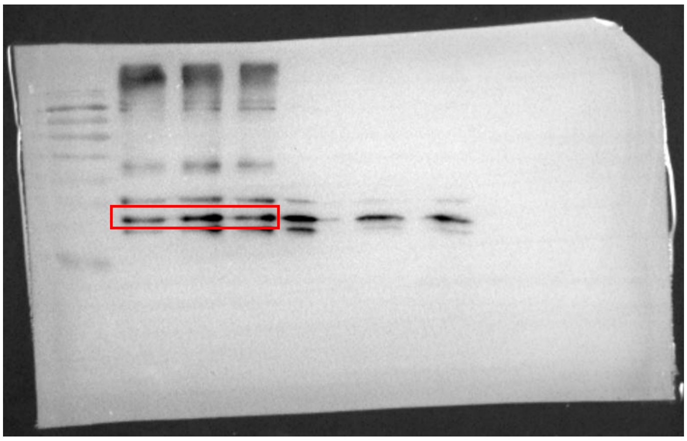

3. Input: HA-Ub-WT

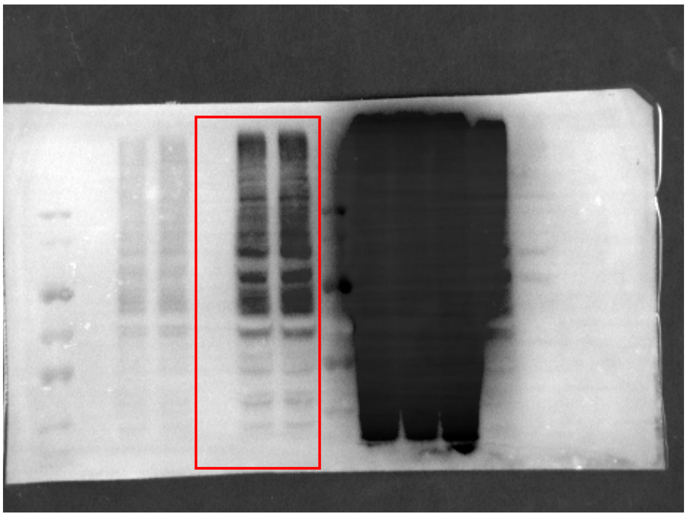

4. Input: Flag-ASC

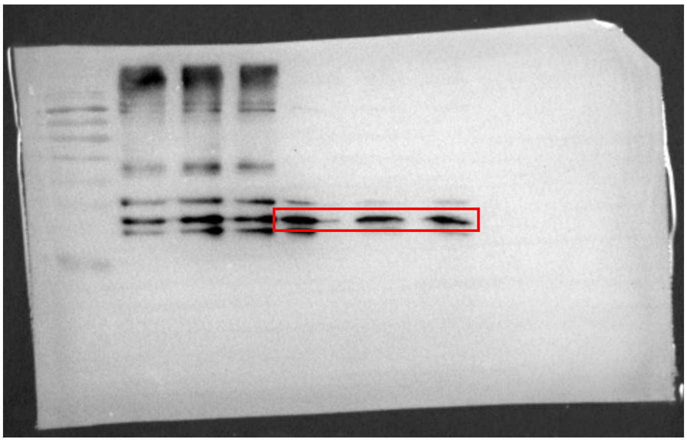

5. Input: Myc-Yod1

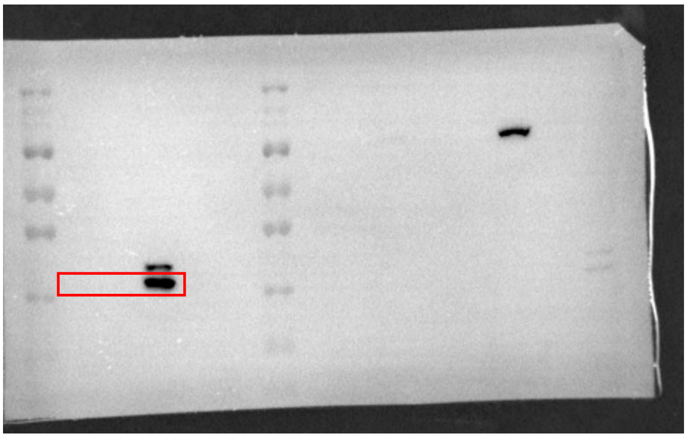

6. ACTIN

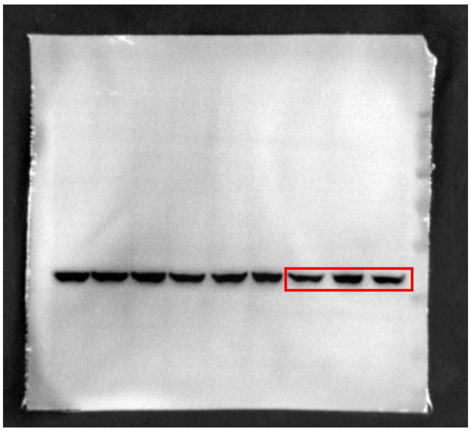

Supplement: Supplementary file 2 — Original Data File [file 41419_2024_6731_MOESM2_ESM.pdf]
